# Defining a Spinal Microcircuit that Gates Myelinated Afferent Input: Implications for Tactile Allodynia

**DOI:** 10.1016/j.celrep.2019.06.040

**Published:** 2019-07-09

**Authors:** Kieran A. Boyle, Mark A. Gradwell, Toshiharu Yasaka, Allen C. Dickie, Erika Polgár, Robert P. Ganley, Desmond P.H. Orr, Masahiko Watanabe, Victoria E. Abraira, Emily D. Kuehn, Amanda L. Zimmerman, David D. Ginty, Robert J. Callister, Brett A. Graham, David I. Hughes

**Affiliations:** 1 Spinal Cord Research Group, Institute of Neuroscience and Psychology, University of Glasgow, Glasgow G12 8QQ, UK; 2 School of Biomedical Sciences and Pharmacy, University of Newcastle, Newcastle NSW 2308, Australia; 3 Department of Anatomy and Physiology, Saga University, Saga 849-8501, Japan; 4 Department of Anatomy, Hokkaido University School of Medicine, Sapporo 060-8638, Japan; 5 Department of Neurobiology, Howard Hughes Medical Institute, Harvard Medical School, Boston, MA 02115, USA

**Keywords:** touch, allodynia, presynaptic inhibition, interneurons, LTMRs, dorsal horn, parvalbumin

## Abstract

Chronic pain presents a major unmet clinical problem. The development of more effective treatments is hindered by our limited understanding of the neuronal circuits underlying sensory perception. Here, we show that parvalbumin (PV)-expressing dorsal horn interneurons modulate the passage of sensory information conveyed by low-threshold mechanoreceptors (LTMRs) directly via presynaptic inhibition and also gate the polysynaptic relay of LTMR input to pain circuits by inhibiting lamina II excitatory interneurons whose axons project into lamina I. We show changes in the functional properties of these PV interneurons following peripheral nerve injury and that silencing these cells unmasks a circuit that allows innocuous touch inputs to activate pain circuits by increasing network activity in laminae I–IV. Such changes are likely to result in the development of tactile allodynia and could be targeted for more effective treatment of mechanical pain.

## Highlights

• Parvalbumin (PV) interneurons mediate presynaptic control of myelinated afferents
• PV interneurons simultaneously mediate postsynaptic inhibition of vertical cells
• Allodynic mice show reduced PV cell excitability but no structural plasticity

## Graphical Abstract

**Figure:**
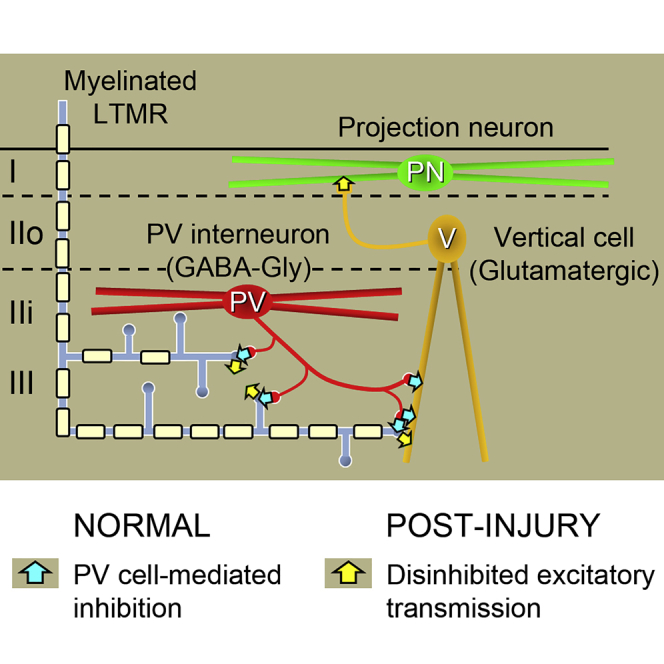

## Teaser

In this study, Boyle et al. identify parvalbumin-expressing spinal interneurons as a principal source of axoaxonic synapses onto cutaneous myelinated afferents and inhibitory inputs onto lamina II vertical cells. Following peripheral nerve injury, disinhibition of these targets facilitates the aberrant recruitment of pain circuits, leading to tactile allodynia.

## Introduction

Chronic pain represents a major global health problem, affecting up to 20% of the adult population (Goldberg and McGee, 2011). One significant obstacle to the development of new therapies is our limited understanding of how neuronal circuits in the spinal cord transmit and modulate sensory information and how changes to these circuits result in altered sensory experience, as seen in chronic pain. A critical component of spinal sensory circuits is the role played by inhibitory interneurons (Todd, 2010, Peirs and Seal, 2016, Moehring et al., 2018), and the loss of spinal inhibition is believed to underlie several forms of chronic pain (Yaksh, 1989, Ahmadi et al., 2002, Moore et al., 2002, Miraucourt et al., 2007). These interneurons comprise a heterogeneous population of cells based on their morphological, electrophysiological, and neurochemical properties and are thought to serve functionally distinct roles (Yasaka et al., 2010, Maxwell et al., 2007). Most studies on spinal inhibition focus on postsynaptic inhibition, involving the release of GABA and/or glycine at axodendritic and/or axosomatic synapses, but GABA release at axoaxonic synapses is also known to mediate presynaptic inhibition of primary afferent central terminals. Although axoaxonic synapses have been described on the central terminals of most types of primary afferents (Réthelyi et al., 1982, Ribeiro-da-Silva and Coimbra, 1982, Todd, 1996, Hughes et al., 2005) and a high incidence of such synaptic connections has been reported in lamina II (Duncan and Morales, 1978), identifying the cells that give rise to these synapses has proven challenging. We have demonstrated that a significant proportion of axoaxonic synapses on the central terminals of myelinated afferents are derived from inhibitory interneurons that express the calcium-binding protein parvalbumin (PV), and that axoaxonic synapses are the predominant form of synaptic output from these cells (Hughes et al., 2012). PV cells have since been shown to play a key role in setting mechanical thresholds in normal and chronic pain states (Petitjean et al., 2015), with the development of nerve-injury-induced tactile allodynia reported to occur in parallel with a reduction of PV-cell-derived postsynaptic inhibitory input to protein kinase C γ (PKCγ) cells. Since the contribution of PV-cell-mediated presynaptic inhibition of low-threshold mechanoreceptive (LTMR) afferents was not considered in this circuit, the aim of our study was to define the principal synaptic targets of inhibitory PV interneurons, identify the primary sources of afferent input to these cells, and establish whether the anatomical or physiological properties of PV cells change with the development of allodynia.

## Results

### PV Cells in Laminae IIi and III Are a Source of Presynaptic Inputs onto Several Classes of Myelinated LTMR Afferent Fibers

**Figure 1:**
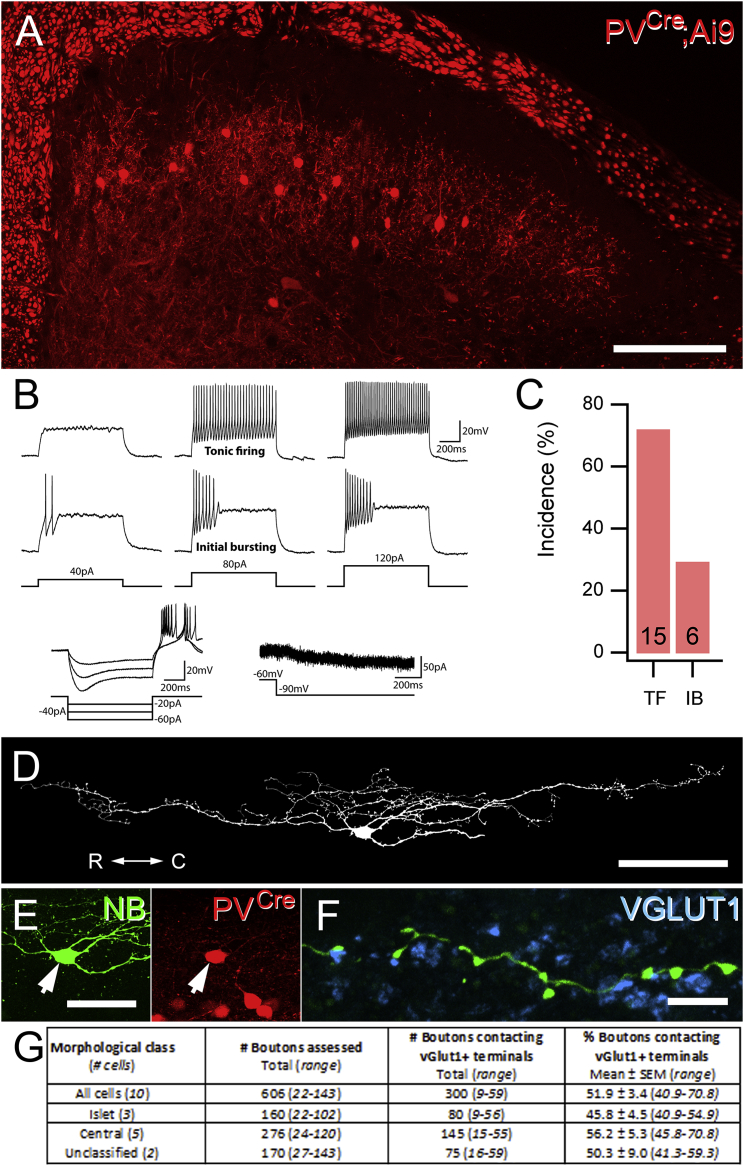
PV Cells in Laminae IIi and III Are a Source of Axoaxonic Contacts onto Myelinated Afferents (A) The expression of tdTom (PV^*Cre*^;Ai9; red) in the spinal dorsal horn of the PV^*Cre*^;Ai9 mouse mirrors the distribution of PV-immunoreactive cells. (B and C) All tdTom cells displayed either tonic firing or initial bursting AP discharge patterns in response to current injection (B, upper traces), as well as a high incidence of the I_*h*_ subthreshold current and associated voltage sag (B, lower traces). Numbers at the base of bars in (C) are the number of cells in each category. (D) NB labeling of recorded neurons shows that most cells displayed islet or central-cell-like morphology (82.3%; 14/17), with the remaining cells being of unclassified morphology. R-C denotes orientation of the rostrocaudal axis. (E) Demonstration of tdTom expression (red) in the cell body of the NB-filled islet cell shown in (D) (NB, green). (F) Several axon terminals in lamina IIi and III derived from this cell (green) contact boutons labeled with VGLUT1 (blue). (G) Table summarizing the incidence of NB-labeled boutons from morphologically defined tdTom-expressing cells in contact with VGLUT1-immunoreactive terminals. Scale bars represent 100 μm (A and D), 25 μm (E), and 5 μm (F).

PV axon terminals have been shown to form axoaxonic synapses onto the central terminals of myelinated afferents (Hughes et al., 2012), but the cells from which these boutons originate have yet to be identified. To clarify this, we carried out targeted whole-cell patch-clamp recordings with Neurobiotin (NB)-filled electrodes from tdTomato (tdTom)-expressing cells in sagittal slices of lumbar spinal cord from a PV^*Cre*^;Ai9 mouse line (Figure 1A). These cells showed either tonic or initial bursting action potential (AP) firing patterns in response to depolarizing current injections and a high incidence of I_*h*_ and/or associated voltage sag in response to membrane hyperpolarization (Figures 1B and 1C; Table S1). Most cells showed islet or central-cell-like morphology, with dendrites elongated in the rostrocaudal axis of the spinal cord (Figure 1D). Detailed analyses of axon from 10 cells revealed that their boutons contain the vesicular GABA transporter (VGAT) and that these often contact axon terminals labeled with vesicular glutamate transporter type 1 (VGLUT1; Figure 1F). VGLUT1 is expressed in axon terminals of both myelinated afferents and corticospinal projections, but only those derived from LTMRs are contacted by multiple VGAT boutons (Todd et al., 2003, Abraira et al., 2017). We found that on average, 51.9% (±3.4%) of boutons in laminae IIi and III from these cells were apposed to VGLUT1-expressing terminals (Figure 1G). While this data identify PV cells in laminae II and III as the source of axoaxonic inputs onto the central terminals of myelinated LTMRs, it also implies that their axons synapse onto dorsal horn neurons.

**Figure 2:**
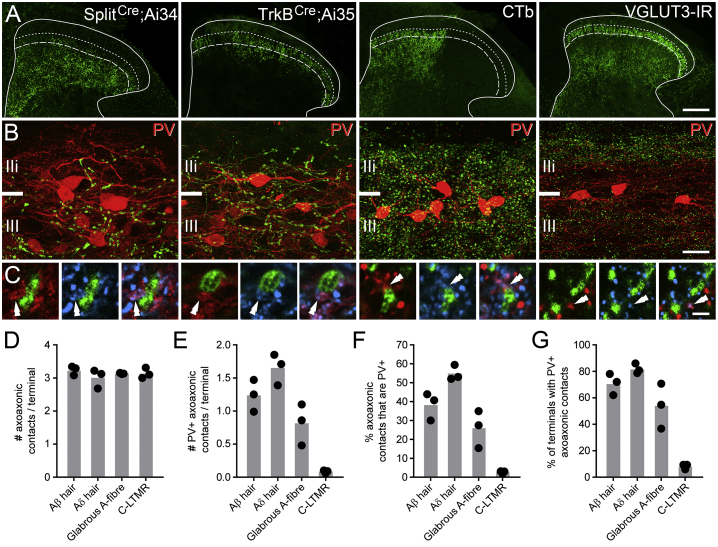
Axoaxonic Contacts from PV Interneurons Target the Central Terminals of Several Classes of Myelinated Afferents (A) The central terminals of Aβ-hair afferents (labeled in the Split^*Cre*^; Ai34 mouse), Aδ-hair afferents (labeled in the TrkB^*CreER*^; Ai35 mouse), myelinated glabrous skin afferents (labeled with CTb), and C-LTMRs (labeled with antibodies to VGLUT3) each display distinctive patterns of arborization (green). (B) The central terminals of myelinated LTMRs overlap extensively with PV cells (red) in laminae IIi and III, whereas C-LTMRs only overlap with the more dorsal aspect of the PV plexus. (C) The central terminals of all classes of LTMRs receive multiple contacts from VGAT boutons (blue); however, only myelinated LTMRs receive extensive input from inhibitory PV terminals (double arrowheads). (D and E) The mean number of VGAT terminals (D) and PV-VGAT terminals (E), respectively, in contact with each class of LTMR afferent. (F and G) The mean percentage of axoaxonic contacts on to each class of afferent that are derived from PV cells (F) and of terminals from each afferent class that have at least one contact from a PV-VGAT bouton (G), respectively. Bars in graphs show means across all animals, and individual points are means of each animal (n = 3 animals per afferent group; 150 terminals analyzed per animal). Scale bars represent 100 μm (A), 20 μm (B), and 2 μm (C).

Recent work has established that virtually all central terminals from myelinated afferent fibers arborizing in the LTMR-recipient zone (LTMR-RZ; laminae IIi–IV) are associated with inhibitory axon terminals (Abraira et al., 2017) and that a significant proportion of these inhibitory inputs express PV. This implies that all LTMRs are under presynaptic control and that many of these axoaxonic synaptic inputs are derived from PV cells. One interpretation of this finding is that axoaxonic synapses from PV cells target only specific classes of LTMR afferents. To address this, we used tissue from Split^*Cre*^;Ai34 and TrkB^*CreER*^;Ai35 mouse lines to label the central terminals of A$\tilde{\beta }$ and Aδ-hair afferents, respectively (Rutlin et al., 2014, Li et al., 2011). We also injected CTb into the glabrous skin of the hindpaw of wild-type mice to label myelinated afferents innervating non-hairy skin, and we used an antibody to VGLUT3 to identify the central terminals of unmyelinated LTMRs (C-LTMRs). We then quantified the incidence of all axoaxonic contacts, including those derived from PV cells, onto the central terminals of each fiber type (Figure 2). These received, on average, three VGAT boutons per terminal (Figures 2C and 2D; Table S2). While most myelinated LTMR axons were apposed to inhibitory PV terminals, C-LTMR terminals rarely received such inputs (Figures 2C and 2E–2G; Table S2). We therefore conclude that PV-expressing interneurons are a source of presynaptic inputs onto several classes of myelinated LTMRs from both hairy and glabrous skin but rarely target C-LTMRs.

### PV Cells Are Innervated by Aβ- and Aδ-Hair Afferents, as well as Myelinated Afferents from Glabrous Skin

**Figure 3:**
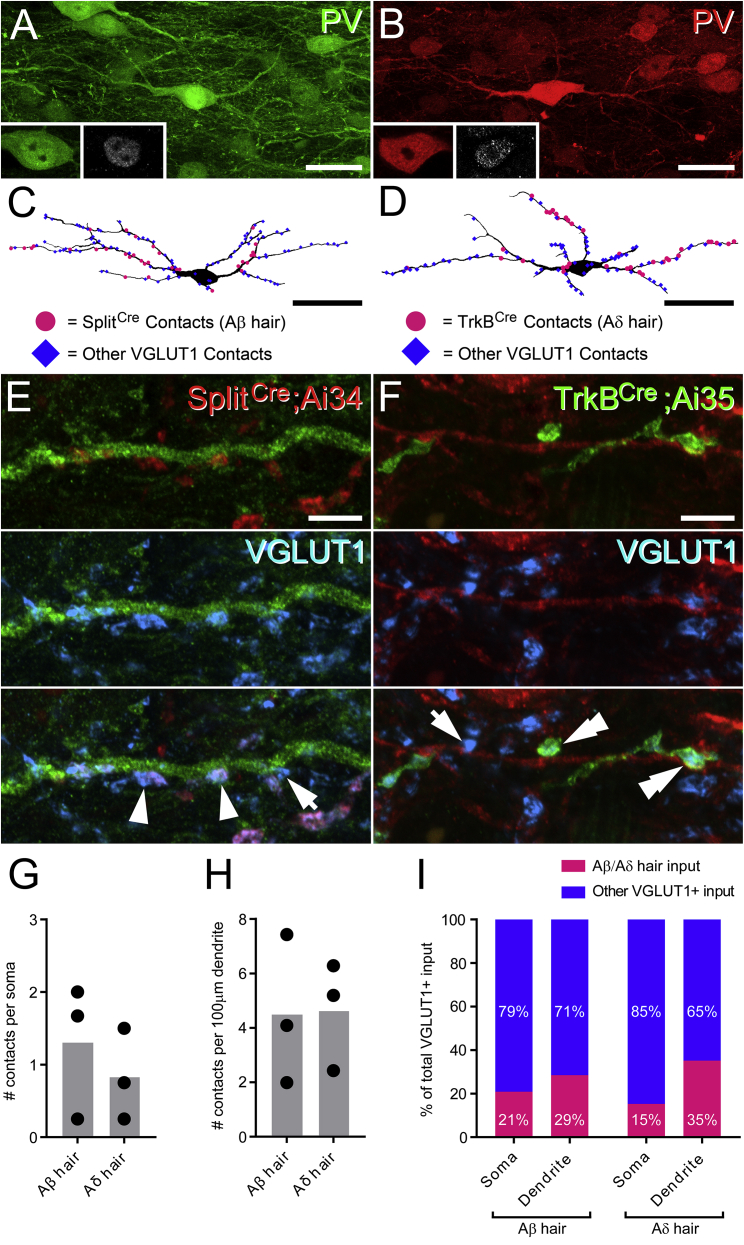
Myelinated Hair Afferents Are a Principal Source of Afferent Input to Inhibitory PV Cells in Laminae IIi and III (A and B) Representative examples of inhibitory PV cells in tissue from Split^*Cre*^; Ai34 (A) or TrkB^*CreER*^; Ai35 (B) mice. Higher magnification insets show the presence of Pax2-immunolabelling (gray) in the nuclei of these cells. (C and D) Reconstructions of the individual inhibitory PV interneurons shown in (A) and (B), respectively, showing the relative positions of contacts from VGLUT1-only (blue diamonds) and Aβ- (C) or Aδ-hair (D) afferent terminals (magenta circles) plotted onto their cell body and dendrites. (E and F) Examples of dendrites from these PV-expressing inhibitory interneurons receiving multiple contacts from axon terminals that either express only VGLUT1 (blue, arrows), tdTom-labeled boutons of Aβ-hair afferents (red; arrowheads in E) derived from Split^*Cre*^;Ai34 mice, or YFP-expressing Aδ-hair afferents (green; double arrowheads in F) from TrkB^*CreER*^;Ai35 mice. (G) Mean number of contacts from Aβ- and Aδ-hair afferent terminals per inhibitory PV soma. (H) Mean number of contacts from Aβ- and Aδ-hair afferent terminals per 100 μm of dendrite of inhibitory PV interneurons. (I) Relative proportion of all VGLUT1 terminals contacting the soma and dendrites of inhibitory PV cells that are derived from Aβ- and Aδ-hair afferents. Bars in (G)–(I) show means across all animals, and individual points show the means of each animal. n = 3 mice per afferent class, with three or four inhibitory PV cells analyzed per mouse. Scale bars represent 25 μm (A and B), 100 μm (C and D), and 5 μm (E and F).

PV cells have been shown to receive synaptic input from myelinated afferent terminals arborizing in the LTMR-RZ (Hughes et al., 2012, Abraira et al., 2017), but we know very little about which classes of myelinated LTMRs innervate inhibitory PV interneurons. To address this, we assessed the incidence of Aβ and Aδ hair LTMR inputs onto inhibitory PV interneurons in laminae IIi and III in tissue from Split^*Cre*^;Ai34 and TrkB^*CreER*^;Ai35 mice using Pax2 immunoreactivity to identify inhibitory PV interneurons (Figures 3A and 3B). The somatodendritic arbors of individual Pax2-expressing PV cells were reconstructed based on PV immunolabeling. All VGLUT1-expressing terminals that apposed the reconstructed neurons were plotted and counted, including those derived from genetically labeled Aβ- and Aδ-hair terminals (Figures 3C and 3D). The dendrites of inhibitory PV cells received a comparable number of dendritic and somatic contacts from both Aβ- and Aδ-hair afferents (∼4 per 100 μm of dendrite and ∼1 per soma). These account for 29% (Aβ) and 35% (Aδ) of all VGLUT1 terminals onto dendrites and 21% (Aβ) and 15% (Aδ) of all VGLUT1 contacts onto soma (Figures 3C–3I; Table S3). In parallel studies, we found that virtually all axon terminals from Aβ- and Aδ-hair or myelinated afferents from glabrous skin that apposed the dendrites of inhibitory PV cells formed excitatory synapses at these sites, as determined by the presence of an intervening Homer punctum (Gutierrez-Mecinas et al., 2016). Together, these findings establish that inhibitory PV interneurons receive rich monosynaptic input from several classes of myelinated LTMRs derived from both hairy and glabrous skin.

### Axons from PV Cells Mediate Two Distinct Forms of Inhibition and Target Several Populations of Lamina II Interneurons, Including Vertical Cells

Our anatomical studies on NB-filled tdTom cells identify the central terminals of myelinated LTMR afferents as the principal synaptic targets of inhibitory PV cells, although they are also known to synapse with the dendrites of both PV and non-PV cells in lamina IIi (Hughes et al., 2012, Petitjean et al., 2015). Vertical cells are one population that are likely to receive synaptic input from both inhibitory PV interneurons and LTMR afferents since their dendrites branch extensively in laminae II and III (Grudt and Perl, 2002, Lu and Perl, 2005, Yasaka et al., 2014). Most of these interneurons are glutamatergic (Maxwell et al., 2007) and often have axon that arborizes in lamina I (Grudt and Perl, 2002, Lu and Perl, 2005, Yasaka et al., 2014) where they synapse onto lamina I neurons, including those projecting to the spinoparabrachial nucleus (Lu and Perl, 2005, Cordero-Erausquin et al., 2009). Since the anatomical features of these cells position them as a potential route for LTMR input into lamina I pain circuits under pathological conditions where spinal inhibition is diminished (Lu et al., 2013, Yasaka et al., 2014), we aimed to determine whether inhibitory synaptic inputs from PV cells are involved in gating LTMR input to vertical cells.

**Figure 4:**
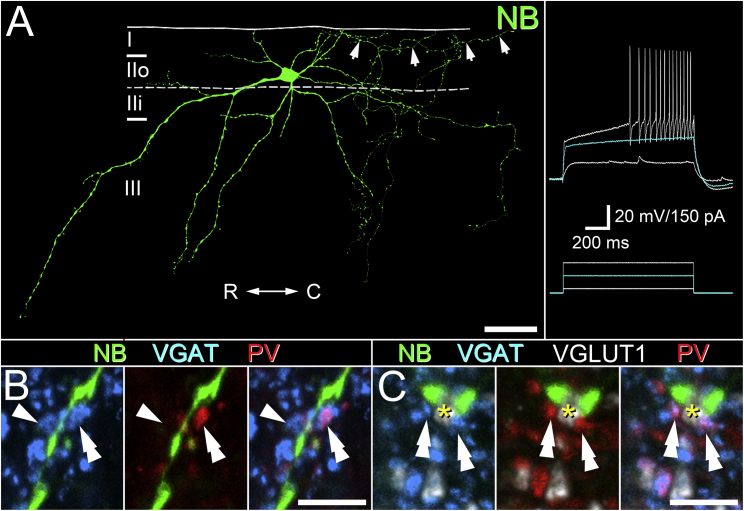
PV Interneurons Are a Source of Inhibitory Inputs to the Dendrites of Vertical Cells and Axoaxonic Contacts to Myelinated Afferents Contacting Those Vertical Cells (A) An example of the characteristic morphology and physiology of vertical cells filled with NB (green) and analyzed in this study. These cells have their cell body in lamina IIo, and most of their dendritic arbor extends into deeper dorsal horn laminae. Only vertical cells that showed delayed- or gap-firing AP discharge patterns in response to current injection (inset) and with axon arborizing in lamina I (arrows) were included in this analysis. R-C denotes orientation of the rostrocaudal axis. (B and C) We assessed the incidence of contacts from VGAT axon terminals (blue) on to vertical cell dendrites in laminae IIi and III (green). In these laminae, the dendrites of vertical cells receive multiple contacts from both VGLUT1 axon terminals (gray, asterisk in C) and VGAT-IR boutons (blue; arrowhead in B). Many of the VGAT boutons are derived from PV cells (red; double arrowheads in B and C), and these inhibitory PV boutons often appose VGLUT1 axon terminals (gray, asterisk in C) that contact the same vertical cell and potentially form triadic synaptic arrangements. Scale bars represent 20 μm (A), 5 μm (B, D, and E), and 50 μm (C).

We carried out blind whole-cell patch-clamp recording of lamina II neurons from spinal cord slices to label vertical cells, as no definitive neurochemical marker is currently known that selectively defines this population (Sathyamurthy et al., 2018, Häring et al., 2018). To be included in this anatomical analysis, each recorded neuron had to display electrophysiological and anatomical features consistent with those described previously for vertical cells; specifically, we looked for delayed-firing AP discharge patterns in response to current injections and a defining morphology featuring a cone-shaped pattern of dendritic branching that extends in a ventral direction (Grudt and Perl, 2002, Yasaka et al., 2010, Yasaka et al., 2014). Only vertical cells with axon that arborized in lamina I were analyzed (Figure 4A). The total number of VGAT-, PV-VGAT-, and VGLUT1-expressing boutons in contact with vertical cell dendrites that arborized in laminae IIi and III was determined in five NB-filled vertical cells. The cumulative length of vertical cell dendrite analyzed was 6,608.7 μm (mean, 1,321.7 μm; range, 792.1–2,423.4 μm). The number of VGAT contacts onto the dendrites in these laminae was 253 terminals per cell (±16.7; range, 196–299), of which 27.7% ± 1.2% were derived from PV cells (Figure 4B). We found that 13.1% ± 1.9% of all VGAT contacts onto vertical cell dendrites also apposed VGLUT1 terminals that contacted vertical cell dendrites (Figure 4C), and these associations are likely to form triadic synaptic connections commonly associated with central terminals of myelinated afferents (Todd, 1996, Watson et al., 2002). Of these putative triadic arrangements, most (61.8% ± 3.7%) were derived from PV cells, and these account for 29.2% ± 4.4% of all PV-VGAT boutons that contact vertical cell dendrites. The mean number of VGLUT1 terminals found in contact with vertical cell dendrites was 59.2 ± 7.9%, of which 96.1% ± 1.8% were defined as being derived from primary afferents based on their direct association with multiple VGAT contacts (Abraira et al., 2017). Most of these myelinated afferent terminals (62.5% ± 7.6%) were in contact with a PV-VGAT bouton, whether in a triadic arrangement with the labeled vertical cell or not. These anatomical arrangements imply that individual PV interneurons have the capacity to mediate two distinct forms of inhibition, presynaptic inhibition of LTMR afferents and postsynaptic inhibition of vertical cell dendrites.

**Figure 5:**
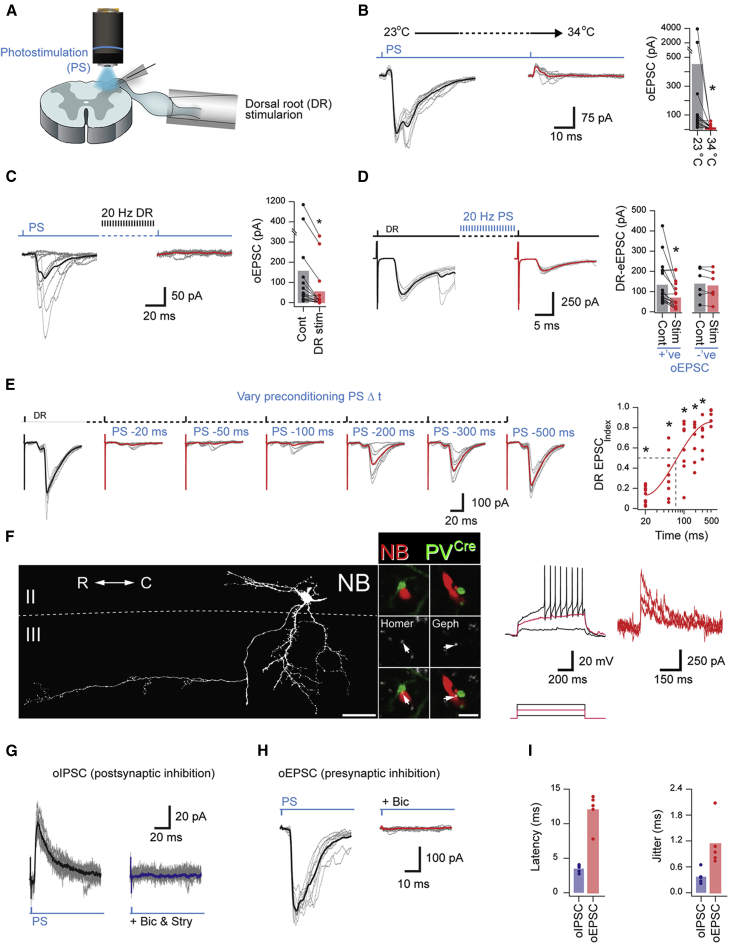
PV Cells in PV^*Cre*^;Ai32 Mice Mediate Both Light-Evoked Presynaptic and Postsynaptic Inhibition (A) Schematic showing the recording setup. (B) PV-photostimulation-evoked oEPSCs (left) that were reduced and/or abolished at an elevated temperature (right; n = 15; 4 mice) as highlighted by group data plots. (C) PV-photostimulation-evoked oEPSCs (left) were abolished by a conditioning stimulus to fatigue primary afferent synapses (right, 1 s DR stimulation at 20 Hz) as highlighted by the group data plot (n = 18; 8 mice). (D) DR-eEPSC amplitude (left) is reduced after a conditioning photostimulation of PV cells (1 s photostimulation at 20 Hz) to fatigue the presynaptic inhibitory synapse (right). This effect was limited to cells that exhibited an oEPSC (left plot; n = 16; 8 mice), but not in cells where no oEPSC was observed (right plot; n = 6; 5 mice). (E) DR-eEPSCs recorded before (black trace) and after (red traces) preconditioning PV cell photostimulation delivered at varying intervals (1-ms pulse, −20 ms to −500 ms). DR-eEPSC amplitude is diminished at short preconditioning intervals (−20 ms to −100 ms) but approximates the baseline response in the preconditioning −500-ms trial. Data were fitted with a Boltzmann function, yielding a half recovery time of 62.5 ms (right; n = at least 5 for each time point; 2 mice). (F) Morphology of a recorded vertical cell (gray), filled with NB. Insets show examples of YFP-expressing PV terminals (green) making excitatory (Homer; gray) and inhibitory synapses (gephyrin; gray) on to the dendrites of the recorded cell (arrows). R-C denotes rostrocaudal axis orientation. Recorded vertical cell displayed delayed AP discharge during depolarizing current step injections (left; lower, 20-pA steps), and A-type potassium currents during a voltage step protocol (right; −100 mV to −40, −30, and −20 mV, respectively). (G and H) Representative PV-photostimulation evoked oIPSCs (G; strychnine and bicuculline sensitive) and oEPSCs (H; bicuculline sensitive) recorded from vertical cells (n = 5; 4 mice). (I) Plots show oIPSC latency and jitter are low, consistent with a monosynaptic connection, whereas longer latency and higher jitter for oEPSCs are consistent with a polysynaptic circuit. ^∗^p < 0.05 by paired t test. Scale bars represent 50 μm (D) and 2 μm (insets).

To determine whether the PV-cell-derived contacts onto both LTMR inputs and vertical cells represent functional synapses, we performed *ex vivo* optogenetic experiments in spinal cord slices from PV^*Cre*^;Ai32 mice (Figure 5A). Presynaptic inhibition has traditionally been difficult to assess directly, as it represents the activation of axoaxonic synapses, leading to GABA-mediated primary afferent depolarization (PAD). Under *in vivo* conditions, PAD inhibits release of glutamate from afferent central terminals; however, when the temperature is lowered, PAD can evoke glutamate release from these terminals, producing excitatory postsynaptic potentials (EPSPs) in motor neurons (Eccles and Willis 1963). This phenomenon can also be detected *in vitro*, with photostimulation of ChR2-expressing GABAergic interneurons also causing temperature-dependent optically evoked excitatory postsynaptic currents (oEPSCs) between proprioceptive afferents and motor neurons (Fink et al., 2014). Here, we have adopted the same approach to study axoaxonic synapses from PV interneurons onto the central terminals of cutaneous afferents. PV-cell-mediated oEPSCs could be reliably recorded at room temperature (23°C) but were abolished by elevating recording bath temperature to 34°C (EPSC_index_; 0.24 ± 0.06; p < 0.001, paired t test, n = 15; 4 mice; Figure 5B). The necessity for afferent function to generate polysynaptic oEPSCs was also tested using high-frequency dorsal root stimulation (1 ms at 20 Hz) to fatigue afferents (Figure 5C). Under these conditions, oEPSCs were significantly reduced and in many cases abolished, confirming the involvement of afferent terminals (EPSC_index_; 0.36 ± 0.09, p < 0.001, paired t test, n = 15; 8 mice).

Using a similar approach, high-frequency photostimulation of PV cells was capable of fatiguing PAD-evoked neurotransmitter release and reducing dorsal root evoked EPSC amplitude (EPSC_index_: 0.53 ± 0.09, p < 0.001, paired t test, n = 16; 8 mice; Figure 5D). Importantly, dorsal root evoked EPSC amplitudes were unaffected by high-frequency photostimulation in cells that lacked oEPSCs (EPSC_index_: 0.91 ± 0.07, p = 0.247, paired t test, n = 6; 5 mice; Figure 5D), suggesting their afferent input was not “gated” by PV-cell-mediated presynaptic inhibition. The duration of PV-cell-mediated presynaptic inhibition was also tested by varying the timing of a single PV-photostimulation pulse preceding dorsal root stimulation (Figure 5E). Prior PV activation and the resulting presynaptic inhibition caused a prominent time-dependent reduction in electrically evoked excitatory postsynaptic current (eEPSC) amplitude, which lasted up to 500 ms (EPSC_index_: 0.12 ± 0.09, p < 0.001; 0.32 ± 0.23, p < 0.001; 0.60 ± 0.27, p = 0.004; 0.66 ± 0.20, p = 0.004; 0.70 ± 0.16, p = 0.007; 0.91 ± 0.07, p = 0.051 for preceding PV + interneuron (IN) photostimulation −20, −50, −100, −200, −300, and −500 ms, respectively, paired t test; n = 9, 8, 8, 7, 6, and 5; 2 mice). Therefore, we can attribute the suppression of dorsal root (DR)-evoked responses to the PV-mediated presynaptic inhibition.

In a subset of experiments, the morphology of recorded cells was recovered (n = 36), and five were subsequently classified as vertical cells (Figure 5F). All vertical cells showed A-type potassium currents (five out of five) and received polysynaptic inward oEPSCs (membrane potential −70 mV), indicative of afferent input gated by presynaptic inhibition (Figure 5H). These responses were abolished by bicuculline (oEPSC_index_; 0.08 ± 0.02; p < 0.001, paired t test, n = 5; 4 mice), consistent with the role for GABA_A_ receptors in presynaptic inhibition and PAD (Eccles et al., 1963). When membrane potential was adjusted to −40 mV, PV photostimulation caused short-latency optically evoked inhibitory postsynaptic currents (oIPSCs) that were abolished by bicuculline and strychnine (Figure 5G). These oIPSCs exhibited short latencies (∼4 ms) and low-onset jitter (∼0.5 ms; Figure 5I) consistent with monosynaptic PV cell input. This contrasts the PAD-evoked oEPSCs, which had longer latencies (∼12 ms) and greater onset jitter (∼1.2 ms; Figure 5I). Together, these findings provide functional confirmation that PV cells provide powerful, convergent inhibition of vertical cells via both direct postsynaptic inhibition as well as presynaptic inhibition of myelinated afferent drive.

### PV Cells Do Not Undergo Significant Structural Changes following Peripheral Nerve Injury

**Figure 6:**
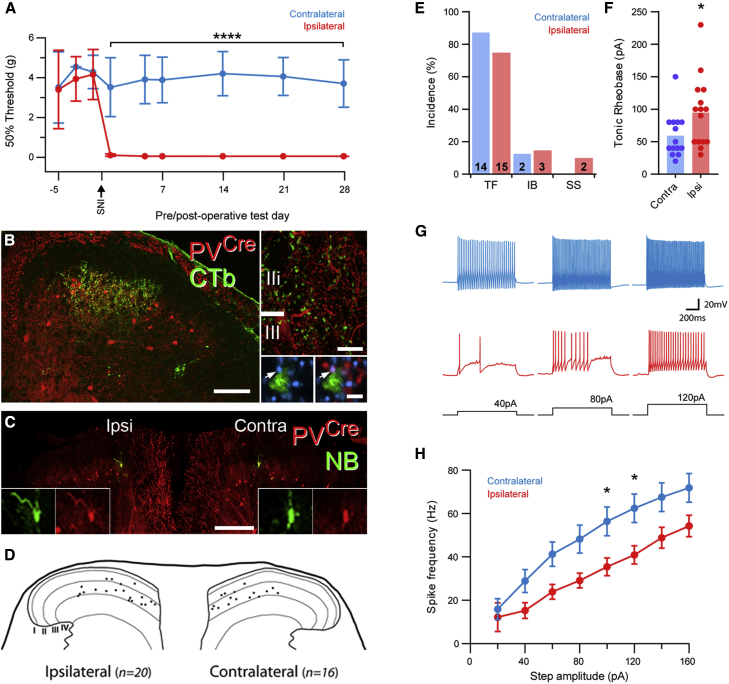
Anatomical and Electrophysiological Features of PV Cells in Allodynic Mice (A) PV^*Cre*^;Ai9 mice that have undergone unilateral SNI (n = 11) develop pronounced punctate tactile allodynia in the skin region innervated by the sural nerve during the first postoperative week, which persists throughout the test period (^∗∗∗∗^p < 0.0001 for contralateral versus ipsilateral at all post-surgery time points, two-way ANOVA with Sidak’s post-test of multiple comparisons). (B) CTb was injected into the glabrous skin region innervated by the sural nerve (ipsilateral to the nerve injury) to label the myelinated afferents that evoke the tactile allodynia (n = 3 animals). The central terminals of these afferents (green) overlap extensively with the plexus of tdTom-expressing PV cells (red) in laminae IIi and III, and receive multiple contacts from VGAT boutons (blue), many of which are derived from PV cells (arrow). (C) Targeted whole-cell patch-clamp recordings from tdTom cells (red) were made in spinal cord slices both ipsilateral (n = 20) and contralateral (n = 16) to the nerve injury and within the central territories of the tibial and common peroneal nerves. NB (green) was included in the recording electrode for post hoc confirmation of tdTom expression in recorded cells. (D) Plot of the relative positions of all cells recorded from the contra- and ipsilateral sides. (E) The incidence of AP firing patterns in tdTom cells is similar on both the contra- and ipsilateral sides, with the exception of two single-spiking neurons that are seen ipsilateral to the nerve injury. Numbers at the base of bars are number of cells in each category. (F) The tonic rheobase is significantly higher in tdTom neurons ipsilateral to nerve injury (^∗^p < 0.05 by unpaired Student’s t test; bars in graph are means from all cells, and individual data points from each cell are overlaid; n = 14 cells contralateral, 15 ipsilateral). (G) Example traces of AP output in response to current injection from tonic-firing cells on the contra- and ipsilateral sides. (H) Input and/or output relationship of tonic-firing tdTom neurons, demonstrating a significantly reduced firing frequency in response to 100 and 120pA current injection on the ipsilateral side (^∗^p < 0.05 by two-way ANOVA with Sidak’s post-test of multiple comparisons; data are shown as mean ± SEM; n = 14 cells contralateral, 15 ipsilateral). Scale bars represent 100 μm (B and C); insets, 10 and 2 μm, respectively.

Given the behavioral evidence implicating PV cells in mechanical allodynia (Petitjean et al., 2015), we aimed to determine whether the anatomical and electrophysiological properties of these cells were altered in mice that had undergone the spared nerve injury (SNI) model of neuropathic pain (Decosterd and Woolf, 2000) and had developed mechanical hypersensitivity (Figure 6A). We first aimed to determine whether peripheral axotomy resulted in a loss of PV interneurons in denervated regions of the spinal dorsal horn. We focused specifically in regions where axotomized afferents from the tibial and common peroneal nerve terminate, as any central changes resulting as a consequence of peripheral nerve injury would most likely be evident here. The expression of prostatic acid phosphatase (PAP) immunolabeling is depleted in injured afferents, and we used this to map the somatotopic representation of afferents from the axotomized tibial and common peroneal nerves (Figures S2A and S2B). We found a total of 214 tdTom cells (range, 41–73) in the denervated area ipsilateral to the nerve injury, and 184 (range, 34–62) in the corresponding regions of the contralateral dorsal horn (n = 4 mice, two sections analyzed per mouse). This equates to a mean of 53.5 ± 6.9 cells per animal ipsilateral to SNI versus 46.0 ± 6.7 on the contralateral side; these means did not differ significantly (p = 0.32 by paired t test; Figure S2C). Therefore, in agreement with previous findings (Petitjean et al., 2015), we find no evidence of a loss of PV cells in denervated regions of the spinal cord after peripheral nerve transection.

Peripheral axotomy has been shown to result in a change in the glomerular appearance of central terminals from non-peptidergic C-fibers and a loss of axoaxonic synapses (Castro-Lopes et al., 1990, Bailey and Ribeiro-da-Silva, 2006). We aimed to address whether similar structural changes occurred on the central terminals of myelinated afferents from skin territories showing heightened mechanical sensitivity following SNI. To label these afferents, we injected CTb into glabrous skin innervated by the sural nerve of three PV^*Cre*^;Ai9 mice that showed mechanical hypersensitivity to static and/or punctate stimulation (Figure 6A). The resulting pattern of CTb labeling in the spinal cord was restricted to the central one-third of the dorsal horn in mid-L5 segment (Figure 6B). We found that 53.3% ± 0.9% of these CTb-labeled terminals in lamina IIi received contacts from VGAT boutons that also expressed tdTom (100 CTb-labeled terminals per animal). The mean number of VGAT boutons in contact with the CTb-labeled terminals was 3.8 ± 0.01, and the average number of VGAT boutons that expressed tdTom was 0.7 ± 0.02. These PV-boutons account for 17.7% ± 0.4% of the total VGAT contacts onto CTb-labeled central terminals. Control experiments, where CTb was injected into the glabrous skin of naive mice, were conducted in parallel (n = 3 animals). The majority of CTb-labeled central terminals from myelinated afferents innervating glabrous skin in naive animals also received contacts from VGAT and PV boutons (54.0% ± 9.8%; 150 boutons per animal). These were associated with 3.1 ± 0.01 VGAT boutons, of which 0.8 ± 0.2 expressed PV. In these control animals, PV boutons account for 25.9% ± 5.7% of the total VGAT contacts onto CTb-labeled central terminals. The differences seen in the mean number of tdTom and VGAT boutons in contact with individual CTb terminals, the mean percentage of CTb terminals in contact with tdTom and VGAT boutons, and the mean percentage of axoaxonic contacts that express tdTom between control and SNI groups were not statistically significant (p = 0.47, 0.95, and 0.22, respectively; unpaired t tests).

It has been reported that the number of tdTom boutons forming inhibitory synapses onto the cell bodies of PKCγ cells in lamina II of PV^*Cre*^;Ai14 mice decreased after peripheral nerve injury (Petitjean et al., 2015). Since this study focused on the cell bodies of PKCγ cells specifically, we determined the incidence of inhibitory PV-cell-derived synapses onto both the somata and dendrites of PKCγ cells in naive mice and those that had undergone SNI 4 weeks earlier (Figure S3). For analysis of sections from nerve-injured animals, only cells in denervated regions, determined by the depletion of PAP immunolabeling in adjacent sections, were included. We found no significant differences between naive and SNI mice in the incidence of inhibitory PV inputs onto PKCγ cell bodies (p = 0.76 by unpaired t test of means and p = 0.9996 for Kolmogorov-Smirnov test of cumulative distribution; Figures S3F and S3G; Table S4) or in the proportion of total somatic inhibitory input onto PKCγ cells derived from PV boutons (p = 0.65 by unpaired t test; Table S4). The incidence and distribution of all other non-PV inhibitory inputs to the soma were also unchanged following SNI (p = 0.96 by unpaired t test of means and p = 0.9888 for Kolmogorov-Smirnov test of cumulative distribution; Figures S3I and S3J; Table S4). The dendritic arbors of a subset of PKCγ cells from each animal were partially reconstructed, and the incidence of inhibitory synaptic inputs (including those derived from PV interneurons) onto these was also compared. There were no significant differences between naive and SNI mice in either the density of PV inhibitory synapses onto PKCγ cell dendrites (p = 0.40, unpaired t test; Figure S3H) or in the proportion of total inhibitory synapses derived from PV cells (p = 0.29, unpaired t test; Table S4). The density of dendritic inhibitory inputs from boutons lacking PV was also unchanged between naive and SNI mice (p = 0.81, unpaired t test; Figure S3K). The average dendritic length reconstructed did not differ significantly between the two groups (p = 0.93 by unpaired t test; Table S4). Taken together, these data suggest that structural changes in synaptic connectivity are unlikely to contribute significantly to the loss of PV-cell-mediated inhibition implicated in the development of mechanical hypersensitivity following peripheral nerve injury.

### Peripheral Nerve Injury Reduces the Excitability of Spinal PV Cells

We then aimed to determine whether the physiological properties of PV neurons differ in mechanically hypersensitive PV^*Cre*^;Ai9 mice following SNI. Whole-cell patch-clamp recordings targeting tdTom cells in transverse spinal cord slices were restricted to the regions corresponding to tibial and common peroneal nerve territories (Figure S2A). We recorded from 20 cells in axotomized regions of the ipsilateral dorsal horn and 16 cells in corresponding locations of the contralateral (intact) dorsal horn (Figures 6C and 6D). Cells from the contralateral dorsal horn displayed similar electrophysiological properties to those recorded in naive PV^*Cre*^;Ai9 mice, showing predominantly tonic-firing discharge patterns in response to current injection and I_*h*_ currents (Figures 6E–6G; Table S1). Furthermore, the passive membrane properties of cells recorded from the ipsilateral and contralateral dorsal horns did not differ (Table S1). The incidence of AP discharge patterns was also broadly similar between the two sides (Figure 6E), but significant differences were seen in the AP discharge properties of those PV cells capable of repetitive firing in the ipsilateral dorsal horn. The amplitude of current injection needed to maintain tonic firing for the entire stimulus in tonic-firing PV cells (which we term the “tonic rheobase”) was significantly higher ipsilateral to the SNI when compared to those in the contralateral dorsal horn (59.3pA ± 9.1 contralateral versus 94.7pA ± 13.9 ipsilateral; p = 0.045 by unpaired Student’s t test; Figures 6F and 6G; Table S1). In addition, the current-frequency relationship for AP discharge was altered in tonic-firing PV cells, with discharge frequency in response to 100- and 120-pA current injection significantly lower on the ipsilateral side (contralateral versus ipsilateral = 56.4 ± 6.6 Hz versus 35.4 ± 4.1 Hz at 100 pA, 62.4 ± 6.6 Hz versus 40.9 ± 4.2 Hz at 120 pA; p = 0.042 and 0.034, respectively, by two-way ANOVA with Sidak’s post-test for multiple comparisons; Figures 6G and 6H; Table S1). These results show that PV cells within the denervated dorsal horn territory have reduced excitability across a number of measures when compared to PV cells in the intact contralateral dorsal horn. Such a shift would likely result in a reduction of PV-cell-mediated inhibition.

### Silencing Neurotransmission in Spinal PV Cells Results in Aberrant Patterns of LTMR Afferent Input Processing in the Dorsal Horn

**Figure 7:**
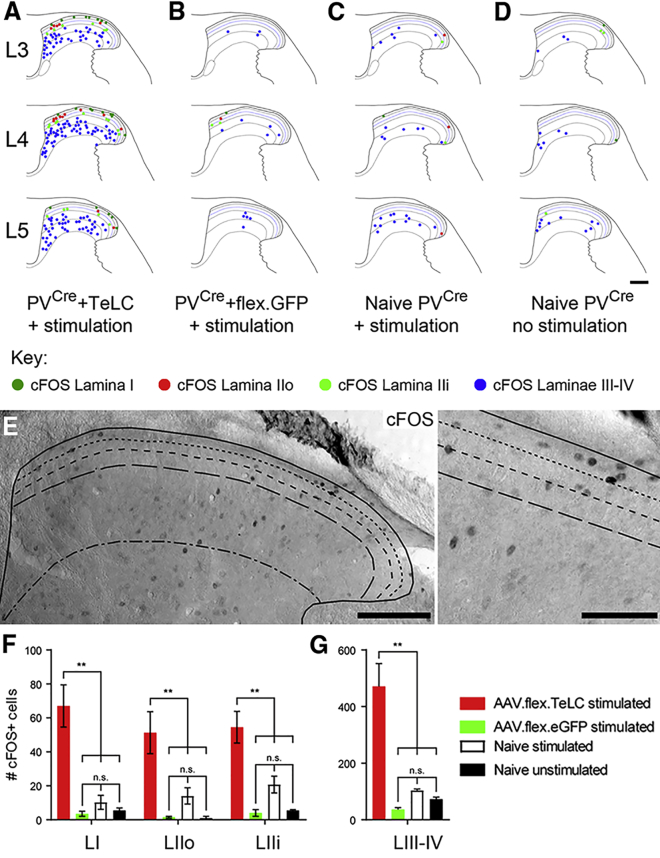
Silencing PV Interneurons with AAV.flex.TeLC Results in Increased Network Activity in Laminae I–IV following Innocuous Tactile Stimulation (A–D) Plots of the distribution of cFOS-labeled cells in laminae I–IV following brush and punctate stimulation of hairy and glabrous skin over the hindpaw and lower limb of PV^*Cre*^ mice that had undergone unilateral intraspinal injections of AAV.flex.TeLC (TeLC; A) or AAV.flex.GFP (B) into lumbar segments L3–L5, naive PV^*Cre*^ mice (C) that had undergone the same hindpaw stimulation, and naive PV^*Cre*^ mice not subjected to the hindpaw stimulation protocol (D). The location of individual cFOS cells in representative sections from each experimental group is depicted, with cells in each lamina denoted by colored circles: lamina I (dark green); IIo (red), IIi (bright green); III and IV (blue). (E) Representative section from a TeLC-treated mouse following the stimulation protocol. Cells are widely distributed across the dorsal horn, with a high incidence of cells in superficial laminae. (F and G) The incidence of cFOS cells is significantly higher in TeLC animals (red) than in any of the control groups, both in the superficial (F) and deeper laminae (G). The incidence of cFOS labeling in control animals did not differ significantly between groups (n.s., p > 0.05; ^∗∗^p < 0.01; one-way ANOVA with Tukey’s post-test of multiple comparisons). Scale bars represent 187 μm (A–D), 100 μm (E), and 50 μm (F).

The significance of inhibition mediated by PV interneurons in setting mechanical thresholds has been established. Selective ablation of spinal PV interneurons leads to the development of mechanical hypersensitivity, whereas the chemogenetic activation of these cells in allodynic mice restores normal mechanical thresholds (Petitjean et al., 2015). However, the route through which LTMR input activates spinal pain circuits remains poorly understood. One hypothesis proposes that a loss of spinal inhibition leads to the polysynaptic activation of lamina I pain projection neurons (Torsney and MacDermott, 2006, Keller et al., 2007), with vertical cells being identified as a likely route through which LTMR input is relayed (Lu et al., 2013). Data generated from our anatomical and electrophysiological experiments support this idea and implicate PV cells as a central component in gating LTMR input to lamina I via this route. To address this more directly, we aimed to determine whether silencing PV cells resulted in altered network activity of lamina I neurons in response to innocuous tactile stimulation. We used unilateral intraspinal injection of an adeno-associated virus (AAV) coding for Cre-dependent expression of tetanus toxin light chain (TeLC) to selectively block synaptic transmission from PV cells in the L3–L5 segments (Foster et al., 2015) and looked for the expression of cFOS as a marker of cell activation following simultaneous unilateral hindpaw stimulation of hairy and glabrous skin LTMRs. In all animals injected with AAV.flex.TeLC, innocuous peripheral manipulation produced robust cFOS immunolabeling throughout laminae I–IV of the ipsilateral dorsal horn, with 10.6% ± 1.3% and 7.6% ± 0.9% of these cells being found in lamina I and lamina IIo, respectively (Figures 7A and 7E). In contrast, cFOS immunolabeling was rarely seen in laminae I and II of control animals with functionally intact PV neurons (AAV.flex.EGFP-injected stimulated mice, naive stimulated mice, and naive unstimulated mice; Figures 7B–7D). The incidence of cFOS cells did not differ between any of the control groups within any of the lamina divisions analyzed (lamina I, IIo, IIi and laminae III–IV; p > 0.05 for all comparisons, one-way ANOVA with Tukey’s test for multiple comparisons; Figures 7F and 7G). In contrast, a significant increase in the incidence of cFOS cells was observed in AAV.flex.TeLC-injected mice compared to all control groups and across all lamina divisions (p < 0.01 for all comparisons, one-way ANOVA with Tukey’s test for multiple comparisons; see Figures 7F and 7G). These findings demonstrate that PV-cell-mediated inhibition plays a crucial role in gating LTMR-evoked recruitment of lamina I neurons.

## Discussion

Our study demonstrates that inhibitory PV interneurons in laminae IIi and III of the spinal dorsal horn are a major source of axoaxonic inputs onto the central terminals of myelinated LTMRs from both hairy and glabrous skin. We also show that these interneurons gate the passage of LTMR afferent input both by GABA-mediated presynaptic inhibition and by inhibiting the postsynaptic targets of these afferents through the release of GABA and glycine. The postsynaptic targets of LTMRs under inhibitory control from PV-expressing interneurons include vertical cells. Under normal circumstances, PV-cell-mediated inhibition of these cells, and of their LTMR input, is likely to play a central role in segregating LTMR afferent input from pain circuits. However, the anatomical features of vertical cells position them to act as a potential route for LTMR input into lamina I, where, under pathological conditions, the loss of PV-cell-mediated inhibition could unmask this relay circuit, leading to the polysynaptic activation of pain circuits. We demonstrate the potency of this pathway by silencing PV cells using viral vectors and showing that innocuous tactile manipulation results in aberrant activation of neurons in laminae I and IIo. Consistent with this model, we also show PV cell excitability is downregulated in a neuropathic model, which would compromise the pre- and postsynaptic inhibitory gating mediated by these cells. Under such circumstances, the aberrant recruitment of vertical cells following LTMR input would help explain the cellular basis of tactile allodynia associated with neuropathic pain.

### PV Interneurons in Laminae IIi and III Are a Source of Axoaxonic Inputs to Myelinated LTMRs

Presynaptic inhibition was first described in group Ia muscle afferents (Frank and Fuortes, 1957, Eccles et al., 1961, Eccles et al., 1962), with anatomical evidence of axoaxonic synapses (presynaptic boutons [P-boutons]) onto these afferent terminals emerging later (Conradi et al., 1983). We identified the source of these P-boutons as a population of interneurons in the deep medial dorsal horn (Hughes et al., 2005), and these have since been shown to contribute to the smooth execution of movement during locomotion (Fink et al., 2014). Axoaxonic synapses have also been described on the central terminals of several classes of cutaneous afferents (Réthelyi et al., 1982, Ribeiro-da-Silva and Coimbra, 1982, Todd, 1996, Watson et al., 2002, Watson, 2004); however, the interneuron populations that give rise to these presynaptic inputs have remained elusive.

Here, we provide direct evidence that axoaxonic synapses onto the central terminals of myelinated afferents in laminae IIi and III arise from local PV interneurons. Most of the PV cells in these laminae are inhibitory interneurons that co-express GABA and glycine (Laing et al., 1994, Abraira et al., 2017), but these have been shown to be distinguishable from the excitatory population by linear discriminant analyses based on morphological features (Abraira et al., 2017). By defining the principal targets of inhibitory PV interneurons as the central terminals of myelinated LTMRs, we conclude that these interneurons are likely to play a direct role in tuning, gating, and prioritizing our responsiveness to the tactile environment (Abraira and Ginty, 2013). The added insight that PV cells regulate LTMR input from glabrous skin, as well as both Aβ- and Aδ-hair afferents from hairy skin, suggests that the loss of pre- and postsynaptic inhibition to these afferents and their synaptic targets is likely to contribute to the development of both the static (punctate) and dynamic forms of allodynia seen clinically (Ochoa and Yarnitsky, 1993, Field et al., 1999, Dhandapani et al., 2018).

### PV Cells Mediate Presynaptic Inhibition of Myelinated LTMRs and Postsynaptic Inhibition of Vertical Cells

Here, we demonstrate that selective activation of a discrete population of dorsal horn inhibitory interneurons gates sensory input from myelinated LTMRs through the simultaneous presynaptic inhibition of the afferents and postsynaptic inhibition of vertical cells. The strength of PV interneuron-mediated presynaptic inhibition in our experiments is emphasized by the ability of a single brief photostimulation to abolish, or substantially reduce (∼90% block), afferent-mediated EPSCs for hundreds of milliseconds, consistent with previous work on presynaptic inhibition in the ventral horn (Eccles et al., 1961, Hubbard and Willis, 1962, Takeuchi and Takeuchi, 1962).

LTMR afferents that are under presynaptic control from PV interneurons are known to innervate several classes of dorsal horn interneurons (Hughes et al., 2012). Here, we show that PV interneurons mediate both presynaptic inhibition of LTMR afferents synapsing onto vertical cells and postsynaptic inhibition of the same vertical cells. Consistent with these observations, we report a high incidence of putative triadic arrangements of inhibitory PV terminals, dendrites of vertical cells, and inputs to these vertical cells from myelinated primary afferent terminals. Synaptic triads are a common feature of boutons that form axoaxonic synapses (Ribeiro-da-Silva et al., 1985, Todd, 1996) and have been shown to involve PV-expressing boutons (Hughes et al., 2012). Our findings show that PV interneurons presynaptically suppress LTMRs while simultaneously inhibiting a postsynaptic target of that same afferent input. From a biological standpoint, this is consistent with the importance of ensuring that innocuous signals are prevented from exciting nociceptive circuits and causing pain. We conclude that both the presynaptic inhibition of cutaneous afferents and the postsynaptic inhibition of vertical cells mediated by PV interneurons have a profound influence in gating the passage of LTMR input in the dorsal horn under normal conditions.

This interpretation is in line with experiments that established PV-cell-mediated inhibition as an important factor in the development of touch-evoked pain-like behaviors in neuropathic mice (Petitjean et al., 2015). This study focused on the PV-mediated inhibition of PKCγ cells and reported a loss of inhibitory PV inputs to these cells after peripheral nerve injury. It was proposed that the resulting disinhibition unmasked a circuit through which LTMR input is relayed through PKCγ cells and ultimately to lamina I. We find no evidence for changes in either the total inhibitory synaptic input to PKCγ cells or the number of inhibitory synaptic inputs derived from PV cells following peripheral nerve injury (see below), but we do find that the incidence of inhibitory inputs, including those derived from PV interneurons, is similar on both PKCγ cells and vertical cells and that inhibitory PV cell excitability is reduced following nerve injury. We reason that if a reduction in PV-mediated inhibition of PKCγ cells under pathological conditions allows LTMR input to be relayed to lamina I, then this would also disinhibit both vertical cells and the LTMR input they receive. This would result in the unmasking of a more direct (disynaptic) route where LTMR input activates lamina I pain circuits and would form the neurological basis of touch-evoked mechanical hypersensitivity.

### Changes in Membrane Excitability, rather than Structural Plasticity, Underlie PV Cell Disinhibition following Peripheral Nerve Injury

In agreement with previous reports, we found no loss of PV cells in dorsal horn regions corresponding to the termination zones of axotomized afferents in the SNI model (Petitjean et al., 2015), but in contrast to this earlier study, we found no change in the incidence of inhibitory PV synapses onto the cell body and dendrites of PKCγ cells. It is possible that this discrepancy is due to subtle differences in the SNI models used, but it remains to be established how sparing afferents from the tibial nerve (Petitjean et al., 2015) results in the loss of PV synapses onto PKCγ cells, whereas sparing afferents from the sural nerve (this study) does not. We also analyzed the incidence of inhibitory PV boutons on the central terminals of intact myelinated sural afferents in allodynic mice and again found no change, implying that axo-axonic synapses also persist. Together with previous data showing that GABA levels, GABA_A_ receptor subunit expression, and VGAT labeling do not change following peripheral axotomy (Polgár and Todd, 2008), we conclude that PV-cell-mediated inhibition is unlikely to be compromised by significant changes in synaptic connectivity, presynaptic transmitter levels, or neurotransmitter receptor expression. We do, however, find significant differences in the excitability of tonic-firing PV cells in denervated territories, with larger current injections required to elicit sustained “tonic” AP discharge and lower discharge frequencies once they are recruited. Earlier studies in a mouse sciatic nerve constriction injury model have reported no difference in the firing patterns of GAD67-EGFP cells in lamina III (Gassner et al., 2013), whereas targeted recordings of lamina II cells identified impaired excitatory drive (Leitner et al., 2013). These observations differ to what we see in the PV interneurons, but this may be reconciled by our targeted study of a more restricted population. Indeed, it was reported that only 3% of lamina III cells recorded in the experiments studying firing patterns expressed PV (Gassner et al., 2013). Consistent with our findings, lamina II islet cells, which are likely to include PV cells, show decreased membrane excitability and altered firing patterns in a rat model of chronic pain (Balasubramanyan et al., 2006). This supports our proposal that a change in neuronal excitability alone, irrespective of any associated structural changes, is sufficient to reduce GABAergic tone centrally, producing a loss of inhibition and resulting in the development of a chronic pain state (Laird and Bennett, 1992, Moore et al., 2002, Baba et al., 2003).

Our finding that TeLC-mediated silencing of PV interneurons increases network activity in both laminae IIo and I after innocuous mechanical stimulation provides additional support for this proposal. The pattern of cFOS labeling in these mice implies that lamina I pain circuits and vertical cells are recruited when PV interneurons are silenced. This is in line with previously described models of dorsal horn processing (Lu and Perl, 2005, Torsney and MacDermott, 2006, Keller et al., 2007, Yasaka et al., 2014) and serves to demonstrate the importance of PV-cell-mediated inhibition in segregating LTMR input from pain circuits. Extending this model to the level of regional sensory processing, the PV cells we have recorded in the SNI model will also have lost most, if not all, afferent input, further compromising the recruitment of these interneurons. This reduction (or loss) of afferent induced (feed-forward) inhibition within the denervated regions is likely to have relevance beyond the denervated zone. Specifically, the axonal and dendritic arbors of PV cells are substantial, with many expanding across somatotopically distinct boundaries (Swett and Woolf, 1985, Takahashi et al., 2003). This configuration positions PV interneurons to provide inhibition to spinal circuits of adjacent skin territories (commonly referred to as “surround inhibition”) and, by extension, allows peripheral-nerve-injury-related disinhibition to also impact sensory processing in adjacent (intact) skin territories, leading to allodynia.

### Conclusions

Recent studies implicating distinct spinal circuits in the development of mechanical hypersensitivity focus solely on the loss, or impairment, of postsynaptic inhibition (Lu et al., 2013, Duan et al., 2014, Foster et al., 2015, Petitjean et al., 2015, Peirs et al., 2015). Collectively, they serve to demonstrate that several distinct circuits are likely to contribute to these chronic pain states, and this level of complexity reflects the difficulty we face in developing effective treatments. Although the aberrant recruitment of vertical cells to relay information to lamina I pain circuits is a prominent feature common to many of these models (Peirs and Seal, 2016, Moehring et al., 2018), the modulation of afferent input from mechanically hypersensitive skin regions has been overlooked.

Our study highlights the importance of both pre- and postsynaptic inhibition arising from PV interneurons in the processing of mechanosensory information. Furthermore, the microcircuit we describe provides a direct route for the relay of LTMR input to lamina I that is normally under strong inhibitory control. This connectivity highlights how PV-interneuron-mediated inhibition helps segregate LTMR and pain circuits for normal sensory perception but can also produce allodynia when these connections fail under pathological conditions. By extension, our work identifies both inhibitory PV interneurons and vertical cells as potential targets for restoring normal sensory processing following the development of tactile allodynia.

## STAR★Methods

### Key Resources Table

**Table:** 

REAGENT or RESOURCE	SOURCE	IDENTIFIER
**Antibodies**		

Goat anti-cFOS (1:500)	Santa Cruz Biotechnology Inc., CA, USA.	Cat#sc-52-G; RRID: AB_2629503
Goat anti-CTb (1:5000)	List Biological Laboratories Inc., CA, USA.	Cat#703; RRID: AB_2314252
Rabbit anti-dsRed (1:1000)	ClonTech Labs Inc., CA, USA.	Cat#632496; RRID: AB_10013483
Mouse anti-Gephyrin (1:2000)	SynapticSystems, Göttingen, Germany.	Cat#147021; RRID: AB_2232546
Chicken anti-GFP (1:1000)	Abcam plc., UK.	Cat#ab13970; RRID: AB_300798
Rabbit anti-Homer1 (1:2000)	Frontier Institute Co. Ltd, Hokkaido, Japan.	Cat#Homer1-Rb-Af1000; RRID: AB_2571774
Chicken anti-Prostatic acid phosphatase (1:1000)	Aves Labs. Inc., OR, USA.	Cat#PAP; RRID: AB_2313557
Goat anti-Parvalbumin (1:500)	SWANT, Bellinzona, Switzerland.	Cat#PVG-214; RRID: AB_2313848
Guinea pig anti-Parvalbumin (1:500)	Frontier Institute Co. Ltd, Hokkaido, Japan.	Cat#PV-GP-Af1000; RRID: AB_2336938
Rabbit anti-PAX2 (1:1000)	Invitrogen; Thermo Fischer Scientific, UK.	Cat#71-6000; RRID: AB_2533990
Rabbit anti-PKCγ (1:1000)	Santa Cruz Biotechnology Inc., CA, USA.	Cat#sc-211; RRID: AB_632234
Guinea pig anti-PKCγ (Af350) (1:1000)	Frontier Institute Co. Ltd, Hokkaido, Japan.	Cat#PKCg-Rb-Af350; RRID: AB_2571826
Goat anti-VGAT (1:1000)	Frontier Institute Co. Ltd, Hokkaido, Japan.	Cat#VGAT-Go-Af620; RRID: AB_2571623
Mouse anti-VGAT (1:1000)	SynapticSystems, Göttingen, Germany.	Cat#131002; RRID: AB_887871
Guinea pig anti-VGLUT1 (1:5000)	Millipore, Chemicon International, UK.	Cat#AB5905; RRID: AB_2301751
Guinea pig anti-VGLUT3 (1:100)	Frontier Institute Co. Ltd, Hokkaido, Japan.	Cat#VGluT3-GP-Af920; RRID: AB_2571856

**Bacterial and Virus Strains**		

AAV1.flex.TeLC-FLAG	Dr. Hendrik Wildner & Prof. Hanns Ulrich Zeilhofer	Foster et al., 2015
AAV8.flex.eGFP	Viral Vector Facility, University of Zurich	v158

**Experimental Models: Organisms/Strains**		

Mouse: Ai9: B6.Cg-Gt(ROSA)26Sor^tm9(CAG-tdTomato)Hze^/J	Prof. Hongkui Zheng; available from The Jackson Laboratory	JAX: 007909
Mouse: Ai32: B6;129S-Gt(ROSA)26Sor^tm32(CAG-COP4∗H134R/EYFP)Hze^/J	The Jackson Laboratory	JAX: 012569
Mouse: Ai34: B6;129S-Gt(ROSA)26Sor^tm34.1(CAG-Syp/tdTomato)Hze^/J	The Jackson Laboratory	JAX: 012570
Mouse: Ai35D: B6;129S-Gt(ROSA)26Sor^tm35.1(CAG-aop3/GFP)Hze^/J	The Jackson Laboratory	JAX: 012735
Mouse: PV^Cre^: B6;129P2-Pvalb^*tm1(cre)Arbr*^/J	Prof. Sylvia Arber; available from The Jackson Laboratory	JAX: 008069
Mouse: TrkB^CreER^: B6.129S6(Cg)-Ntrk2^tm3.1(cre/ERT2)Ddg^/J	Prof. David Ginty; available from The Jackson Laboratory	JAX: 027214
Mouse: Split^Cre^	Prof. David Ginty	Rutlin et al., 2014

**Software and Algorithms**		

Neurolucida	MBF Bioscience, VT, USA	https://www.mbfbioscience.com/neurolucida
Neurolucida Explorer	MBF Bioscience, VT, USA	https://www.mbfbioscience.com/neurolucida-explorer
pClamp	Molecular Devices, CA, USA	https://www.moleculardevices.com/products/axon-patch-clamp-system/acquisition-and-analysis-software/pclamp-software-suite#gref
AxoGraph X	Dr. John Clements	https://axograph.com/
Zen Black	Carl Zeiss, Germany	https://www.zeiss.com/microscopy/int/products/microscope-software/zen.html
Prism	GraphPad Software, CA, USA	https://www.graphpad.com/scientific-software/prism/
InStat	GraphPad Software, CA, USA	https://www.graphpad.com/scientific-software/instat/

### Lead Contact and Materials Availability

Further information and requests for resources and reagents should be directed to and will be fulfilled by the Lead Contact, Dr. David I. Hughes (David.I.Hughes@glasgow.ac.uk).

### Experimental Model and Subject Details

All mice were bred in house at the University of Glasgow (UoG), University of Newcastle (UoN), Harvard Medical School (HMS), or Saga University (SU). All experimental procedures conducted at UoG were performed in accordance with the European Community directive 86/609/EEC and UK Animals (Scientific Procedures) Act 1986. All experimental procedures carried out at UoG, UoN, HMS and SU were approved by local Animal Care and Ethics Committees, and conducted in accordance with local guidelines. All experiments were carried out on adult mice of either sex (body weights 20-30 g). Mice used in experiments had not previously had any drug treatments or procedures performed on them, unless otherwise stated. In all cases, experiments were conducted in wild-type C57Bl6 mice, or transgenic lines derived by crossing fluorescently-labeled mouse reporter lines expressing Ai9 (RCL-tdTomato; Stock number 007909), Ai32 (Channelrhodopsin-2/YFP, Stock number: 012569), Ai34 (RCL-synaptophysin/tdTomato; Stock number 012570), or Ai35D (RCL-Arch/GFP; Stock number 012735) from Jackson Laboratory, with PV^*Cre*^ (B6;129P2-Pvalb^*tm1(cre)Arbr*^/J, from Jackson Laboratory, Stock number 008069; Petitjean et al., 2015), Split^*Cre*^ or TrkB^*CreER*^ lines, respectively (provided by Prof DD Ginty; see Rutlin et al., 2014, Li et al., 2011). Cre recombinase expression in TrkB^*CreER*^;Ai35 mice was activated using administration of the transgene-inducing agent tamoxifen (intraperitoneal injection of 1mg tamoxifen at p14; Rutlin et al., 2014).

### Method Details

#### Targeted whole-cell patch-clamp recordings *in vitro*

Spinal cord slices from wild-type, naive and nerve-injured PV^*Cre*^;Ai9 mice were prepared using previously described techniques (Graham et al., 2011). Briefly, animals were anaesthetized with ketamine (100 mg kg−1 I.P.) or isoflurane and decapitated. The lumbosacral enlargement of the spinal cord was exposed using a ventral approach and rapidly removed, then placed in ice-cold sucrose substituted artificial cerebrospinal fluid (ACSF) containing (in mM): 250 sucrose, 25 NaHCO_2_, 10 glucose, 2.5KCl, 1 NaH_2_PO_4_, 1 MgCl_2_ and 2.5 CaCl_2_. Transverse or parasagittal slices (from L3–L5 segments; 300 μm thick) were obtained using a vibrating blade microtome (Leica VT-1000S, Heidelberg, Germany, or Microm HM650V, Fisher Scientific) and then transferred to an interface incubation chamber containing oxygenated ACSF (118 mM NaCl substituted for sucrose). In some experiments, parasagittal or transverse slices were prepared with intact dorsal roots attached (Torsney and MacDermott, 2006). For transverse slices prepared from nerve-injured mice, a notch was made with a pair of 25-guage needles on the edge of the ventral horn contralateral to the nerve injury to allow identification of the contralateral and ipsilateral sides of the cord once the slice was transferred to the recording chamber. Slices were allowed to equilibrate for 1 h at room temperature (22–24°C) prior to recording. Slices were transferred to a recording chamber and continually superfused (bath volume 0.4 ml; exchange rate 4–6 bath volumes per minute) with ACSF constantly bubbled with Carbonox (95% O_2_ and 5% CO_2_) to achieve a final pH of 7.3–7.4. Recordings were obtained at either room temperature (22–24°C) or elevated bath temperature (32–34°C) as indicated.

Neurons in laminae II and III expressing tdTomato (Ai9 lines) were first identified under fluorescence using either a rhodamine or fluorescein filter set, respectively, and then visualized using near-infrared differential interference contrast optics (IR-DIC) for targeted recordings. In experiments to label vertical cells in wild-type mice, blind whole-cell patch-clamp recordings were made from neurons in the dorsal part of lamina II, as previously described (Yasaka et al., 2010). Recordings were taken using Neurobiotin-filled pipettes (0.2%; Vector Laboratories, Peterborough, UK) with a potassium gluconate-based internal solution containing (in mM): 135 potassium gluconate, 6 NaCl, 2 MgCl_2_, 10 HEPES, 0.1 EGTA, 2 MgATP, 0.3 NaGTP, pH 7.3 (with KOH), as described previously (Hughes et al., 2012). In some cases, an internal solution containing the following was used (in mM): 120 Cs-methylsulfonate, 10 Namethylsulfonate, 10 EGTA, 1 CaCl2, 10 HEPES, 5 QX-314-Cl[2(triethylamino)-N-(2,6-dimethylphenyl) acetamine chloride], and 2Mg^2+^-ATP, pH adjusted to 7.2 with CsOH, osmolarity 290 mOsm (Torsney and MacDermott, 2006). Recordings were established in whole-cell voltage-clamp (holding potential −70 mV) using a Multiclamp 700B amplifier (Molecular Devices, Sunnyvale, CA, USA), digitized online (sampled at 10–20 kHz and filtered at 5–10 kHz), via an ITC-18 computer interface (Instrutech, Long Island, NY, USA) or a Digidata 1440A digitiser (Molecular Devices), and stored on a Macintosh computer using Axograph X software (Kagi, Berkley, CA, USA) or a PC using pClamp software (Molecular Devices). After obtaining the whole-cell recording configuration, series resistance, input resistance and membrane capacitance were calculated based on the response to a 5 mV hyperpolarising voltage step (10 ms duration) from a holding potential of −70 mV. These values were monitored at the beginning and end of each recording session and data were rejected if values changed by more than 30%.

Action potential (AP) discharge was studied in current-clamp recording mode. The membrane potential recorded < 15 s after switching from voltage to current clamp was designated as resting membrane potential (RMP) and subsequent recordings were made from this potential. All reported membrane potential values have been corrected for the liquid junction potential. AP discharge was studied by injecting a series of depolarizing step-currents (800 ms duration, 20 pA increments, delivered every 8 s) through the recording electrode at a membrane potential of −60 to −85 mV (small bias currents of ± 10 pA were sometimes injected to achieve this potential). During this protocol sustained depolarization was limited to −20 mV, in regions of the voltage trace not containing APs, to avoid cell damage. AP discharge was classified according to previously published criteria (Graham et al., 2007, Yasaka et al., 2010). Briefly, tonic firing (TF) is characterized by sustained repetitive AP discharge throughout the depolarising step, initial bursting (IB) is characterized by repetitive AP discharge at the beginning of the current step which subsequently ceases and single spiking (SS) is characterized by the discharge of one or two APs at the onset of the depolarising current step. This classification scheme also identifies delayed firing (DF), where there is a significant delay between the onset of the depolarising step and AP discharge, and reluctant firing (RF), where cells do not discharge APs even at the maximum depolarising current injection tested.

All analyses of AP properties were performed in Clampfit software (Molecular Devices), and for each cell the mean values are reported from two identical protocol runs. Analysis of the frequency of AP discharge in tonic firing and initial bursting cells was performed by detecting APs using a threshold-based method, and the mean instantaneous frequency was calculated for each depolarising current step. Analysis of the characteristics of single APs was performed on the first AP to be elicited at rheobase (the minimum depolarising current required to initiate AP firing). AP threshold was defined as the membrane potential when the derivative of the AP rising phase reached 10 Vs^-1^. AP width was calculated as the time difference between AP threshold on the rising and falling phases of the AP. AP height was defined as the difference between AP threshold and the maximum positive peak, whereas after hyperpolarisation (AHP) amplitude was defined as the difference between threshold and the maximum negative peak following the AP. The latency of discharge was defined as the time between the onset of the depolarising current injection and the first AP threshold. For tonic firing cells, the tonic rheobase was defined as the minimum depolarising current injected which resulted in sustained AP firing for the duration of the current injection.

Voltage ‘sag ratio’ was determined from hyperpolarising current step responses (−20pA increments from a membrane potential of −65mV) as the ratio of the peak amplitude of the negative voltage response over the steady-state response at the end of the step (mean potential of the last 100ms of the hyperpolarising step response). Cells with a sag ratio of ≤ 0.9 were considered to display voltage sag. Subthreshold I_*h*_ current was revealed by a hyperpolarising step from a holding potential of −60mV to −90mV for 1 s. Automated *P/N* leak subtraction was used to remove capacitive and leak currents, and the mean current was measured for the last 50ms of the hyperpolarising step. Cells which displayed an inward current ≥ 5pA (compared to baseline at the −60mV holding potential) were considered to exhibit I_*h*_ current.

#### Photoactivation of PV cells: optogenetic studies ex vitro

The postsynaptic targets of PV neurons were studied in spinal cord slices from PV^*Cre*^;Ai32 mice by recording optically evoked excitatory postsynaptic currents (oEPSCs) and optically evoked inhibitory postsynaptic currents (oIPSCs). Full-field photostimulation (PS) of PV-ChR2-expressing neurons was achieved using single light pulses (470nm wavelength, 1 ms, 15mW) delivered by a preciseExcite CoolLED illumination system, which was collimated and coupled to the epifluorescence path of an Olympus BX51 microscope. All experiments were carried out under a × 40, 0.8 numerical aperture (NA) water-immersion lens. Recordings were undertaken as described above, but targeted to unidentified PV-ChR2 negative neurons within or dorsal to the YFP plexus. This was confirmed by lack of photocurrent during PS in voltage clamp, at a holding potential of −70 mV. Under these conditions all inward PS-evoked currents were mediated by excitatory (CNQX sensitive – 10 μM) synapses. We also tested the bicuculline (10μM) sensitivity of PS-evoked EPSCs as previous work has shown that bicuculline-sensitive PS-evoked EPSCs can arise from inhibitory axoaxonic inputs onto primary afferents, releasing GABA to mediate presynaptic inhibition (Fink et al., 2014). In afferent terminals where a relatively high equilibrium potential of chloride exists (∼-40 mV), this causes primary afferent depolarisation (PAD), which leads, in turn, to synaptic release of glutamate from the afferent terminal (at room temperature). This results in the generation of an oEPSC which can be recorded in the postsynaptic neuron (at room temperature). We used this pharmacology to identify recorded neurons with primary afferent inputs that received presynaptic inhibition regulated by PV-ChR2 neurons. Postsynaptic inhibition mediated by PV-ChR2 neurons was also assessed by adjusting the holding potential to −40mV, which resulted in outward PS-evoked currents that were insensitive to CNQX (10 μM), but could be abolished by co-application of strychnine (1 μM) and bicuculline (10 μM). For temperature-dependence experiments bath temperature was controlled using a TC324B temperature controller (Warner Instruments) and switched between room (23°C) and elevated temperature (34°C) during recordings. Experiments combining PS and dorsal root stimulation used a suction electrode as described above. Strychnine, bicuculline, and CNQX were obtained from Sigma-Aldrich. Group data from experiments assessing time dependence of optically-evoked PV cell-mediated presynaptic inhibition of dorsal root-evoked EPSCs was fitted with a Boltzmann function as follows: I/I_max_ = 1 –1/[1 + exp (V –V1/2)/κ], where I/I_max_ = normalized current, V = membrane potential, V1/2 = voltage at half-maximal activation (or inactivation), and κ is the slope factor.

#### Tissue preparation for immunocytochemistry

For immunocytochemical studies on perfusion-fixed material, mice were overdosed with pentobarbitone (800 mg kg−1, *i.p.*) and perfused transcardially with 4% depolymerized formaldehyde or 4% depolymerized formaldehyde with 0.2% glutaraldehyde, and post-fixed in the same fixative for an additional 2 hours. Transverse or sagittal sections (60 μm thick) from the lumbar enlargement (L3–L5) were cut on a vibrating blade microtome (VT1200 or VT1000S, Leica, Milton Keynes, United Kingdom), and were subsequently incubated in 50% ethanol in phosphate buffer for 30 minutes. Sections were then incubated in cocktails of primary antibodies for 72 h (see Key Resources Table for details), with primary antibody labeling being detected using species-specific secondary antibodies conjugated to Alexa 488, Alexa 647 (both from Molecular Probes Inc., Eugene, OR, USA), Rhodamine Red or Pacific Blue (both from Jackson Immunoresearch Laboratories, West Grove, PA, USA). All antibodies (primary and secondary) used in immunofluorescence protocols were diluted in phosphate buffered saline (PBS) that contained 0.3M NaCl and 0.3% Triton X-100, and incubations were carried out at 4°C. Sections were mounted on glass slides in Vectashield anti-fade mounting medium (Vector Laboratories, Peterborough, UK).

Laser-scanning confocal microscopy was then carried out using either a Bio-Rad Radiance 2100 confocal microscope (Hemel Hempstead, UK) equipped with a krypton–argon laser, or a Zeiss LSM710 confocal microscope with Argon multi-line, 405 nm diode, 561 nm solid state and 633 nm HeNe lasers, scanned through Plan-Apochromat x20, Plan-Apochromat x40/1.3 Oil DIC, or Plan-Apochromat 63x/1.40 Oil DIC M27 lenses, with zoom between 1 and 2, and z-steps ranging between 0.3 and 1 μm. Confocal image stacks were analyzed offline, using Neurolucida and Neurolucida Explorer software (MBF Bioscience, Williston, VT, USA). For image presentation, the tonal range of individual channels was adjusted in projected stacks using Adobe Photoshop 10 (Adobe Systems, San Jose, CA). No adjustments were made to gamma levels. For generation of figures to assess cell morphology, projections were made of confocal image stack mosaics that included the cell body, dendrites and axon of each recorded neurons. These stacks were viewed in Adobe Photoshop 10, where all labelled profiles for each cell were selected and pasted onto a black background, as described previously (Yasaka et al., 2010).

### Surgical procedures and behavioral testing

#### Transganglionic labeling of glabrous skin afferents

To label the central terminals of myelinated glabrous skin afferents, five naive PV^*Cre*^;Ai9 mice were anaesthetised with isoflurane, then 10 μl of 1% cholera toxin B subunit (CTb; product no. C-9903; Sigma-Aldrich, UK) was injected into the glabrous skin overlying the promontory of the tarsus. These localized injections ensured that only glabrous skin afferents (from the tibial and common peroneal nerve territories) were labeled. All animals recovered from the surgery and were perfused transcardially with 4% depolymerized formaldehyde two days later, to allow for transport of CTb into the central terminals of the glabrous skin afferents. Spinal cord tissue was extracted and processed for subsequent anatomical studies as described above.

#### Mouse model of chronic pain: peripheral nerve injury

To study spinal circuitry in allodynic animals, we carried out the spared nerve injury model (SNI; Decosterd and Woolf, 2000) in PV^*Cre*^;Ai9 mice. Specifically, a 2 to 3mm length of the tibial and common peroneal nerves was removed between two tight ligatures with 7-0 Mersilk under general anesthesia. Great care was taken to ensure that the sural nerve was not manipulated. Behavioral responses to mechanical stimulation of skin regions innervated by the sural nerve were tested using von Frey filaments with logarithmically incremental stiffness prior to, and up to 28 day after, surgery. The 50% paw withdrawal threshold was calculated by Dixon’s nonparametric test (Polgár et al., 2005). Two-way ANOVA with Sidak’s post-test of multiple comparisons was used to determine whether there was a significant reduction in withdrawal threshold of the ipsilateral hind-paw in the SNI animals at each time point, with a *p-value* of less than 0.05 being accepted as significant. Mice showing clear, persistent evidence of altered responsiveness to von Frey application were then prepared for either anatomical or electrophysiological studies.

A critical consideration for studying the electrophysiological properties of PV-expressing cells in allodynic mice is confident targeting of recordings to the region of the spinal dorsal horn containing axotomised axons from the tibial and common peroneal nerves. The somatotopic arrangement of sensory fibers from constituent branches of the sciatic nerve in the dorsal horn has previously been described in the rat (Swett and Woolf, 1985), however, no such data is available for the mouse. To determine the spinal distribution of lesioned afferents in the SNI model, we examined the central labeling patterns for prostatic acid phosphatase (PAP) in six PV^*Cre*^;Ai9 mice that had undergone surgery. In naive mice and rats, PAP labels non-peptidergic C-fibers, most of which also express fluoride resistant acid phosphatase (FRAP) and IB_4_, and central labeling for these markers in axotomised afferents is known to be depleted following peripheral nerve injury (Shehab and Atkinson, 1986, Shehab et al., 2004). By immunostaining for PAP in mice that had undergone the unilateral spared nerve injury, we could delineate the central termination patterns for axotomised afferents. Four weeks post-surgery, the animals were fixed by transcardial perfusion with 4% depolymerized formaldehyde. Transverse sections (60 μm thick) from each spinal segment between L2 to S1 were cut on a vibrating blade microtome. For each segment, sections were collected in series into three bottles, from which one bottle from each segment was then processed to reveal immunolabelling for PAP. Sections were incubated in goat anti-PAP (diluted 1:1000) for 72 hours, followed by donkey anti-goat secondary antibody conjugated to Alexa 488 (Jackson ImmunoResearch, diluted 1:500) for 24 hours. All sections were viewed on a Nikon Eclipse E600 Microscope. Bright field images of all sections were taken using an Axiocam 4.8 then arranged in segmental order in accordance with images from Allen Mouse Spinal Cord and Brain Atlas (Allen Institute for Brain science website). The distribution of PAP immunolabelling was then plotted onto corresponding outlines of each spinal segment of the mouse cord using Photoshop CS (Adobe Systems, San Jose, CA), and a composite representative montage was then generated.

For anatomical studies to look at the incidence of inhibitory axoaxonic contacts on to the central terminals of allodynic afferents, four mice underwent injection of 10 μl of 1% CTb into the glabrous skin territory of the sural nerve at twelve days post-nerve injury to specifically label myelinated afferents from skin regions showing hypersensitivity mechanical to punctate mechanical stimulation. These mice were subsequently perfused (two days post injection) for anatomical studies. We also assessed the effect of SNI on the incidence of inhibitory synaptic inputs on to PKCγ-expressing interneurons in regions of the dorsal horn where axotomised afferents terminate. Sagittal sections from the L4 and L5 spinal segments of naive and SNI mice were prepared (n = 3 mice per group). Areas within the denervated regions of SNI mice were identified using the expression of PAP immunolabelling in adjacent sections (see above and Figure S2). Selected sections were stained with a cocktail of antibodies to VGAT, PV, PKCγ and gephyrin. Confocal image stacks of these sections were analyzed to determine the incidence of inhibitory synaptic inputs on to the cell body and dendrites of PKCγ-expressing cells in lamina IIi of naive and SNI mice, and the proportion of inhibitory synaptic inputs derived from PV terminals was also assessed.

The remaining SNI mice were prepared for targeted whole-cell patch-clamp recordings of tdTomato-expressing cells, as described above. In these animals, recordings were only carried out in transverse slices of spinal cord prepared from L3 to L5 spinal segments. To ensure we were recording from cells in appropriate regions of the dorsal horn where axotomised afferents terminate, we only targeted cells the medial half of the caudal L3 segment, the medial two-thirds of the L4 segment, and the medial third of the rostral L5 segment, based on the results generated from mapping the central arbors of afferents from the tibial and common peroneal nerves. Recordings were made in the dorsal horn ipsilateral to nerve injury, and also in corresponding regions of the gray matter from the contralateral dorsal horn for direct comparison.

#### Intraspinal AAV injections for silencing of PV cells

To determine the consequence of silencing the synaptic transmission mediated by PV-expressing cells on the spinal circuits responsive to LTMR afferent input, we injected an adeno-associated virus (AAV) for Cre-dependent expression of tetanus toxin light chain (AAV1.flex.TeLC-Flag; hereafter referred to as AAV.flex.TeLC) into the spinal dorsal horn of adult PV^*Cre*^ mice, as described previously (Foster et al., 2015). Mice were anesthetized with isoflurane and placed in a stereotaxic frame with 2 vertebral clamps attached to the T12 and L1 vertebrae. The spaces between the laminae of T12–T13 and T13–L1 vertebrae were exposed, and a small incision was made in the dura on the right side of the midline in each space. A hole was drilled through the lamina of the T13 vertebra on the right hand side, and an incision was then made through the underlying dura. Intraspinal injections of 300 nL of AAV.flex.TeLC (2 × 10^8^ particles/injection) were made through each of the three incisions in the dura at a depth of 300 μ m below the spinal cord surface and 400 μm lateral to the midline. Injections were made at a rate of 30 nL per min with a 10 μL Hamilton syringe attached to a glass micropipette (inner tip diameter 40 μm) using a syringe pump (Pump 11 Elite; Harvard Apparatus, Holliston, MA). The locations of the three injection sites were chosen to correspond to spinal segments L3, L4 and L5, which receive afferents from most of the lower limb. All animals made an uneventful recovery.

Six days after intraspinal injections, animals were re-anaesthetized with isoflurane, then subjected to unilateral mechanical stimulation (displacement) of hair shafts overlying the right hind-paw and lower limb with camel-hair brush, and simultaneous application of a von Frey filament (filament 2.83, target force 0.07 g; North Coast Medical Inc., Gilroy, CA, USA) to the glabrous skin of the hind-paw, for 2 minutes. Animals were returned to their home-cage upon completion of the stimulation protocol. The mice were then re-anaesthetised and perfused 2 hours after the onset of the mechanical stimulation with 4% depolymerized formaldehyde, as described previously. Control animals that had undergone identical unilateral stimulation, including PV^*Cre*^ mice injected with AAV8.flex.eGFP (hereafter referred to as AAV.flex.eGFP; 2.6 × 10^8^ particles/injection; n = 2), and naive (un-operated) wild-type mice (n = 4), were also set up in parallel. An additional control group of naive (un-operated, unstimulated) wild-type mice were also used (n = 2). Transverse sections (60 μm thick) of lumbar spinal cord sections from L3, L4 and L5 spinal segments were processed for immunocytochemistry, as described above, with the contralateral ventral white matter notched for side recognition. Sections were first incubated 0.03% H_2_O_2_ (to quench endogenous peroxidase activity), then in goat anti-cFOS for 72 hours, biotinylated donkey anti-goat for 24 hours, then Avidin-horseradish peroxidase for 24 hours. Peroxidase labeling was visualized using 3,3′ 5,5′ diaminobenzidine (DAB) as a chromogen. The distribution of cFOS-immunolabelled cells in laminae I-IV were then plotted on to representative templates of L3, L4 and L5 spinal segments (taken from Allen Brain Atlas). Sections were viewed on a Nikon Elipse E600 microscope equipped with a Zeiss Axiocam, using a 40 × oil-immersion lens to determine the extent of labeling. The assessor was blinded to experimental animal group and side of stimulation. Three sections from each spinal segment from each animal were analyzed. The mean incidence of cFOS-immunolabelled cells in laminae I, II outer (IIo), II inner (IIi), III and IV was then compared between the various control groups using one-way ANOVA followed by Tukey’s test for multiple comparisons.

### Quantification and Statistical Analysis

Data are reported in the text as mean values ± the standard error of the mean (SEM), unless otherwise stated. Statistical analyses were performed in Prism or InStat software (GraphPad, San Diego). For comparisons of two groups paired or unpaired Student’s t tests were applied as appropriate. For comparisons of more than three groups with one independent variable, a one–way ANOVA with Tukey’s post-test for multiple comparisons was used, whereas a two–way ANOVA with Sidak’s post-test was used for comparisons of more than three groups with two independent variables. For analysis of von Frey testing in SNI mice, a repeated-measures two–way ANOVA with Sidak’s post-test was applied. Cumulative distributions of PV-positive and PV-negative inhibitory synaptic contacts onto the cell bodies of PKCγ neurons were compared between naive and SNI mice using the Kolmogorov-Smirnov test. ∗p < 0.05, ∗∗p < 0.01, ∗∗∗p < 0.001, ∗∗∗∗P,0.0001. Brief details of statistical tests are also included in figure legends.

## Supplemental Information

Document S1. Figures S1–S3 and Tables S1–S4

Document S2. Article plus Supplemental Information
