# Supplementary material for: Defining a Spinal Microcircuit that Gates Myelinated Afferent Input: Implications for Tactile Allodynia
Source: Cell Rep. 2019 Jul 9;28(2):526–540.e6. doi: 10.1016/j.celrep.2019.06.040 (PMC6635381; doi:10.1016/j.celrep.2019.06.040)
Supplement: Document S1. Figures S1–S3 and Tables S1–S4 [file mmc1.pdf]

Cell Reports, Volume *28*

Supplemental Information

Defining a Spinal Microcircuit

that Gates Myelinated Afferent Input:

Implications for Tactile Allodynia

Kieran A. Boyle, Mark A. Gradwell, Toshiharu Yasaka, Allen C. Dickie, Erika Polgár, Robert P. Ganley, Desmond P.H. Orr, Masahiko Watanabe, Victoria E. Abraira, Emily D. Kuehn, Amanda L. Zimmerman, David D. Ginty, Robert J. Callister, Brett A. Graham, and David I. Hughes

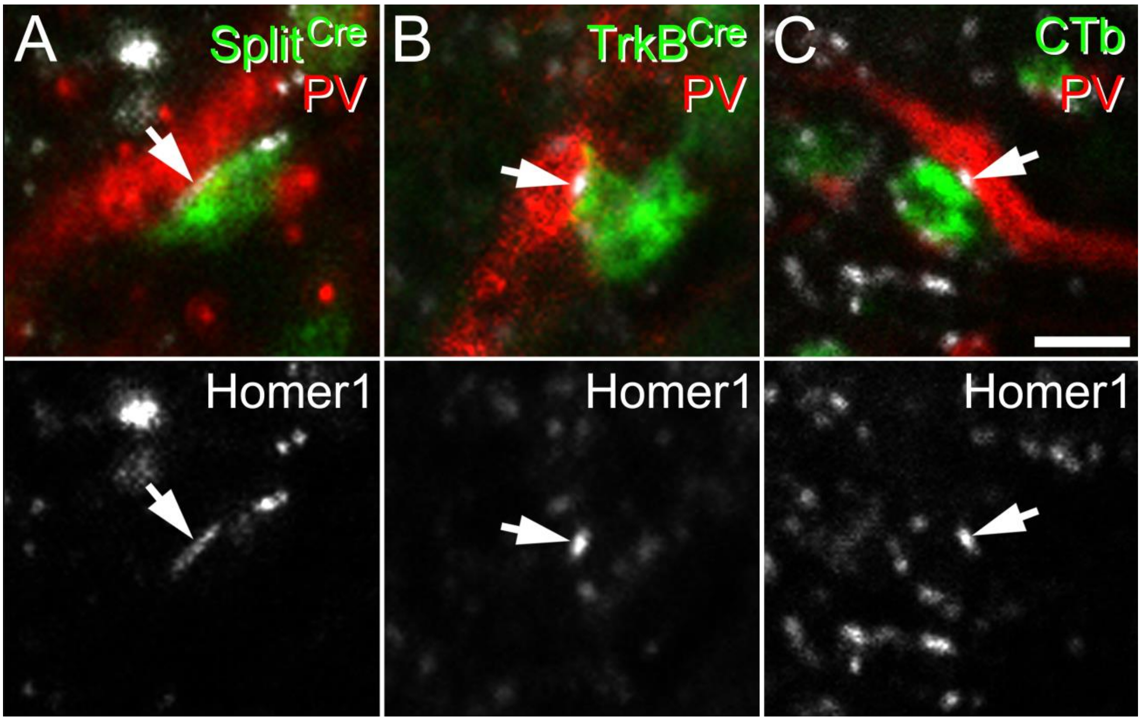

	Aβ hair	Aδ hair	Glabrous A
% of labelled LTMR terminals apposed to Homer punctum	97.5 ± 0.5 (97 & 98)	97.0 ± 0.0 (97 & 97)	99.0 ± 1.0 (98 & 100)

Figure S1 (Relates to Figure 3). Axon terminals of myelinated LTMRs form synapses on to inhibitory PV-expressing cells in laminae IIi and III. The dendrites of inhibitory PV-immunoreactive cells in laminae IIi and III (red; A, B, C), receive multiple contacts from the central terminals of myelinated LTMRs (green) such as those from Aβ hair afferents (labelled in the Split^{Cre};Ai34 mouse; A), Aδ hair afferents (labelled in the TrkB^{Cre};Ai35 mouse; B) and myelinated glabrous skin afferents (labelled with CTb injected into the glabrous skin of the hind-foot; C). Virtually all such contacts (>98%) were associated with a Homer1-immunoreactive punctum (grey), indicating the presence of an excitatory synaptic specialisation. This provides anatomical evidence that myelinated LTMRs provide monosynaptic inputs to inhibitory PV-immunoreactive cells in the spinal dorsal horn. n =2 mice per group. Values in table are presented as mean ± SEM (individual values). Scale bar = 2 μm

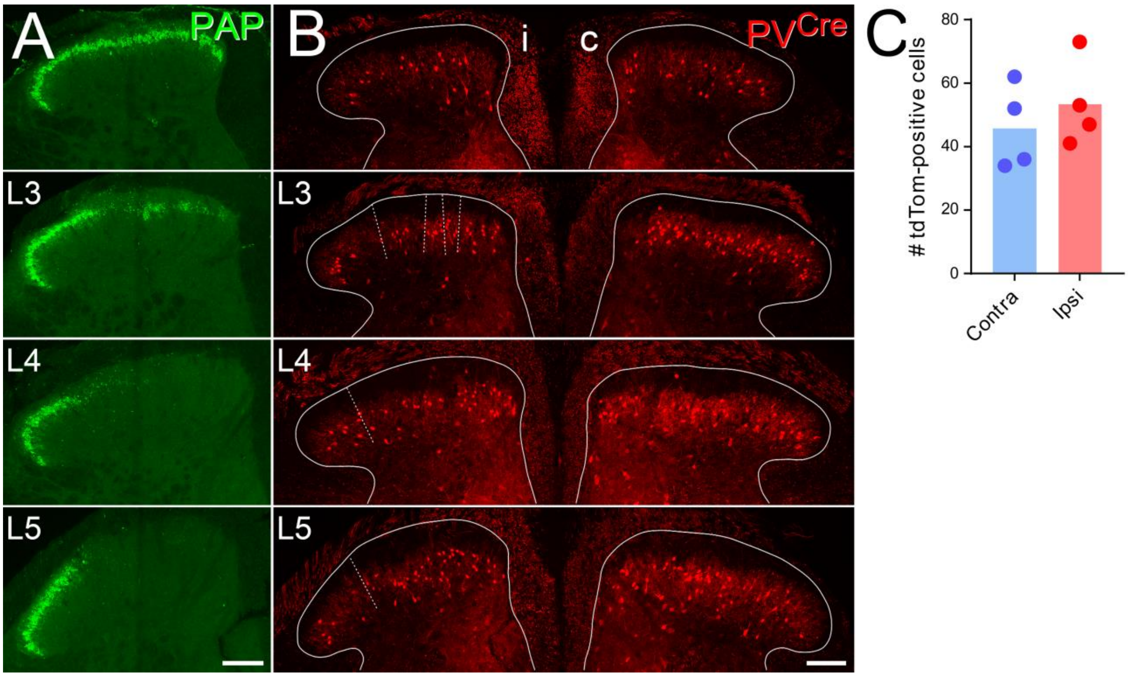

Figure S2 (Relates to Figure 6 and S3). Somatotopic representation of axotomised afferent fibres in the mouse spinal dorsal horn following SNI: the effect of peripheral axotomy on tdTom expression in a PV^{Cre};Ai9 mouse. Prostatic acid phosphatase (PAP) immunolabelling (green) labels the central projections of non-peptidergic C-fibres predominantly, and is visualised as a continuous dense plexus of axon terminals in lamina II in naïve animals. Following peripheral nerve injury, axotomised afferents downregulate PAP, and the depletion of immunolabelling may be used to map the central terminations of lesioned afferents. (A) Using this approach in SNI mice, we find that axotomised axons from the tibial and common peroneal nerves occupy the medial aspect of the dorsal horn, primarily in spinal segments L3 to L5, with maximal depletion occurring from caudal L3 to rostral L5. Here, we show the pattern on PAP-immunolabelling in representative sections from the middle of L2, L3, L4 and L5 spinal segments ipsilateral to the peripheral nerve injury. (B) We used the pattern of PAP-immunolabelling to define regions of the dorsal horn where axotomised afferents terminate (dashed lines), and this allowed us to determine whether peripheral axotomy results in a depletion of tdTom-expressing cells (red) centrally. Sides ipsilateral and contralateral to peripheral nerve injury are denoted with i and c, respectively. (C) We find no evidence for a loss of tdTom-expressing cells in L4 territories of the tibial and common peroneal nerve 4 weeks after peripheral transection (P=0.32 by paired t-test; n = 4 animals, 2 sections analysed per animal; mean number (range) of tdTom cells = 46.0 (34-62) contralateral, 53.5 (41-73) ipsilateral). Scale bars = 100 µm

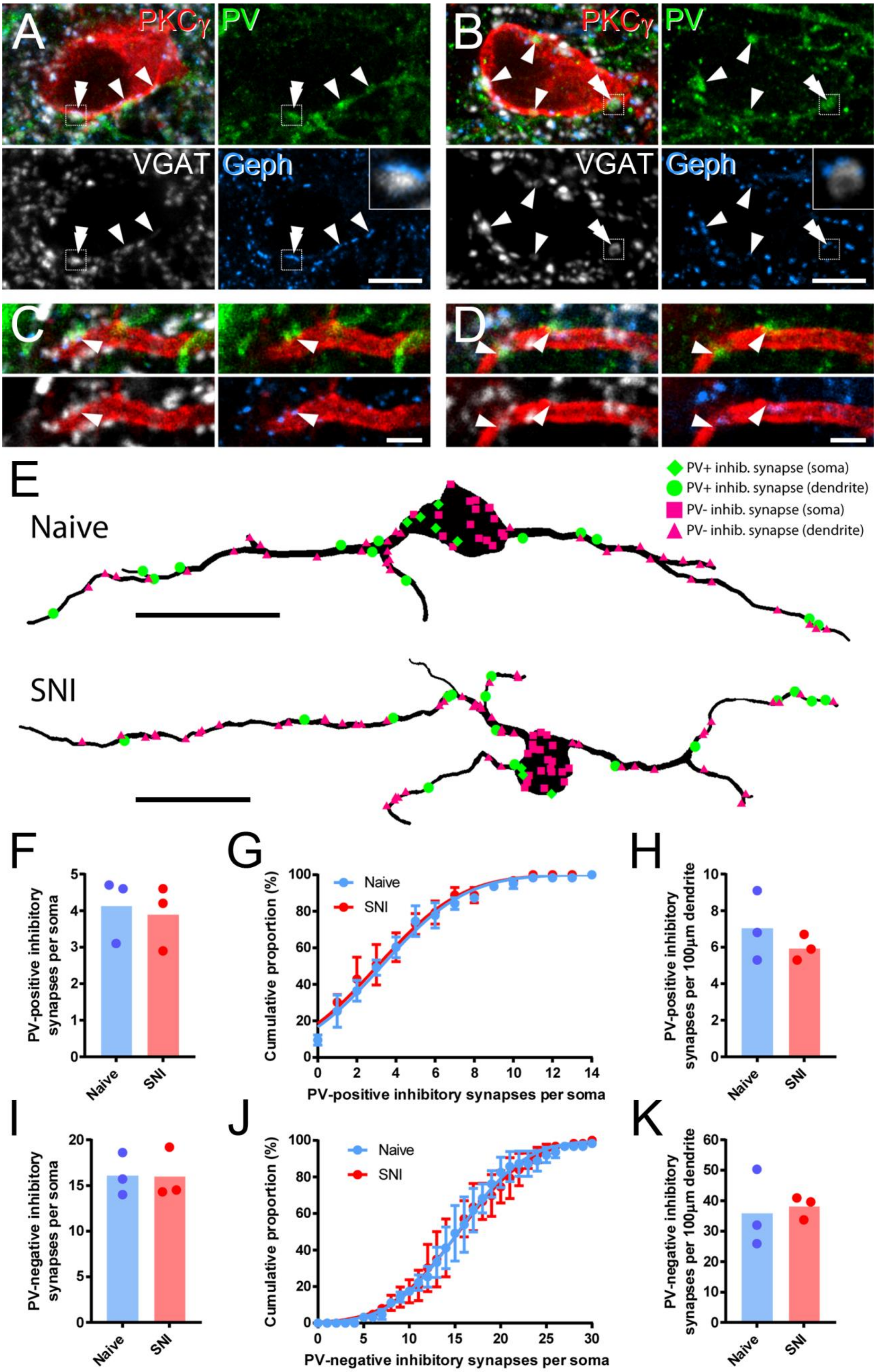

A
PKCγ
PV
VGAT
Geph
B
C
D
E
PV+ inhib. synapse (soma)
PV+ inhib. synapse (dendrite)
PV- inhib. synapse (soma)
PV- inhib. synapse (dendrite)
Naive
SNI
F
PV-positive inhibitory synapses per soma
G
Cumulative proportion (%)
PV-positive inhibitory synapses per soma
H
PV-positive inhibitory synapses per 100µm dendrite
I
PV-negative inhibitory synapses per soma
J
Cumulative proportion (%)
PV-negative inhibitory synapses per soma
K
PV-negative inhibitory synapses per 100µm dendrite

Figure S3 (Relates to Figure 6). Peripheral nerve injury does not result in alterations of inhibitory synaptic inputs to PKCγ cells. We assessed the effect of SNI on the incidence of inhibitory synaptic inputs on to PKCγ-expressing interneurons in regions of the dorsal horn where axotomised afferents terminate. (A and B) Representative images of cell bodies of PKCγ-expressing interneurons (red) from naive mice (A) and SNI mice (B) show the identical patterns of association with profiles immunolabelled for VGAT (grey), PV (green) and gephyrin (blue), with several examples of inhibitory PV boutons forming synaptic inputs on to the PKCγ cells (arrowheads). Insets show the relationship between inhibitory terminals and gephyrin puncta of the outlined examples in more detail. (C and D) The dendrites of PKCγ-expressing interneurons in both naïve (C) and SNI mice (D) receive multiple inhibitory synaptic inputs, with examples of those derived from PV terminals highlighted (arrowheads). (E) To determine the extent of inhibitory input on to the soma and dendrites of PKCγ cells, we reconstructed the morphology of individual cells from naïve and SNI mice then plotted all inhibitory synaptic inputs on to these traces. (F-H) We found no significant differences in the mean number of inhibitory synaptic contacts derived from PV-expressing interneurons per PKCγ soma (F; P=0.76 by unpaired t-test), the distribution of the number of PV interneuron-derived inhibitory synaptic contacts per PKCγ soma (G; P=0.9996 by Kolmogorov-Smirnov test) or the mean number of PV interneuron-derived inhibitory synaptic contacts per 100µm of dendrite (H; P=0.40 by unpaired t-test) between naïve and SNI mice. (I-K) Similarly, we find no significant differences in the mean number of inhibitory synaptic contacts derived from interneurons that do not express PV per PKCγ soma (I; P=0.96 by unpaired t-test), the distribution of the number of these PV-negative inhibitory synaptic contacts per PKCγ soma (J; P=0.9888 by Kolmogorov-Smirnov test) or the mean number of these PV-negative inhibitory synaptic contacts per 100µm of dendrite (K; P=0.81 by unpaired t-test) between naïve and SNI mice. Bars in F, H ,I & K show means from all animals, individual points show means for each animal. Data in G & J are means ± SEM. For analysis of contacts onto PKCγ cell bodies, 3 mice were analysed per group (naïve and SNI), with n=21 cells per mouse (63 PKCγ cells in total). For analysis of contacts onto PKCγ cell dendrites, mean number (range) of PKCγ cells analysed = 7.7 (6-10) for naïve and 7 per mouse for SNI, from 3 mice per group. Scale bars (µm): A and B = 5: C and D = 2; E = 20

**Mouse strain**	**PV^{Cre};Ai9**	**PV^{Cre};Ai9**	**PV^{Cre};Ai9**
	Naïve	SNI (contralateral)	SNI (ipsilateral)
Number of cells analysed	21	16	20
Mean resting membrane potential mV ±SEM	-68.3 ± 1.6	-71.8 ± 2.6	-68.5 ± 2.5
Mean whole-cell capacitance pF ±SEM; (±*SD*)	15.4 ± 1.5	12.8 ± 1.8	15.2 ± 2.0
Mean input resistance MΩ ±SEM	991 ± 116	919 ± 127	856 ± 183
Incidence of firing pattern @ -60 to 65mV *TF* = tonic firing; *IB* = Initial bursting; *SS* = single spiking (*# of cells*)	TF: 71% (*15*)<br>IB: 29% (*6*)	TF: 87.5% (*14*)<br>IB: 12.5% (2)	TF: 75% (*15*)<br>IB: 10% (*3*)<br>SS: 10% (*2*)
Mean rheobase current pA ±SEM	53.8 ± 7.2	38.8 ± 5.6	62.5 ± 14.0
Mean 'tonic rheobase' current pA ±SEM (*# cells*)	68.0 ± 13.0 (*15*)	59.3 ± 9.1 (*14*)	94.7 ± 13.9* (*15*)
Mean tonic firing frequency (at 100pA) Hz ±SEM (*# cells*)	44.4 ± 3.0 (*14*)	56.4 ± 6.6 (*14*)	35.4 ± 4.1* (*14*)
Incidence of voltage sag and/or I_h subthreshold current	87.5% (*14/16 cells tested*)	100.0% (*14/14 cells tested*)	90.0% (*18/20 cells tested*)
Mean I_h amplitude pA ±SEM (*# cells*)	-30.4 ± 13.6 (*8*)	-56.5 ± 19.0 (*7*)	-40.5 ± 10.1 (*12*)
AP thresholds at rheobase pA ±SEM	-42.2 ± 0.6	-46.0 ± 1.2	-44.3 ± 1.4
AP peak amplitude (mV) pA ±SEM	37.2 ± 2.0	48.5 ± 2.7	45.9 ± 2.5
AP base width (ms) ms ±SEM	2.24 ± 0.13	1.95 ± 0.11	2.11 ± 0.19
Peak AHP amplitude (mV) mV ±SEM	-33.4 ± 1.0	-38.5 ± 1.8	-35.0 ± 1.4

Table S1 (Relates to Figure 1 and Figure 6). Summary of the main electrophysiological properties of PV-expressing cells in naïve and allodynic mice. Recordings in naïve animals were made from sagittal spinal cord slices, whereas recordings from SNI animals were made from transverse spinal cord slices to allow direct comparison of cells recorded ipsilateral and contralateral to the lesioned side. *P<0.05 ipsilateral vs. contralateral by unpaired Student's t-test for tonic rheobase and 2-way ANOVA with Sidak's post-test for tonic firing frequency at 100pA current injection.

Primary afferent type	# of axo-axonic contacts/terminal	# of PV axo-axonic contacts/terminal	% of axo-axonic contacts that are PV	% of terminals with PV contact(s)
Aβ hair	3.2 ± 0.1<br>(3.1 – 3.3)	1.2 ± 0.1<br>(1.0 – 1.5)	38.2 ± 4.2<br>(30.0 – 43.9)	70.7 ± 4.7<br>(62.0 – 72.0)
Aδ hair	3.0 ± 0.2<br>(2.7 – 3.2)	1.7 ± 0.1<br>(1.4 – 1.9)	54.8 ± 2.3<br>(52.0 – 59.4)	81.8 ± 2.2<br>(78.7 – 86.0)
Glabrous A-fibre	3.1 ± 0.01<br>(3.12 – 3.15)	0.8 ± 0.2<br>(0.5 – 1.1)	25.9 ± 5.7<br>(15.4 – 35.0)	54.0 ± 9.8<br>(36.7 – 70.7)
C-LTMR	3.2 ± 0.1<br>(3.1 – 3.4)	0.08 ± 0.01<br>(0.06 – 0.10)	2.7 ± 0.4<br>(1.9 – 3.1)	8.2 ± 1.1<br>(6.0 – 9.3)

Table S2 (relates to Figure 2). Parvalbumin interneuron-derived axoaxonic inhibitory input to defined classes of primary afferent fibres. Values are means ± SEM (range) from 150 primary afferent terminals per animal (from 3 mice for each primary afferent type).

		Aβ hair	Aδ hair
Somata	# of contacts / inhibitory PV soma	1.3 ± 0.5 (0.3 – 1.7)	0.8 ± 0.4 (0.3 – 1.5)
	% of total myelinated LTMR input onto inhibitory PV somata	20.9 ± 8.7 (3.6 – 30.2)	15.3 ± 8.2 (4.2 – 31.3)
Dendrites	# of contacts / 100µm inhibitory PV dendrite	4.5 ± 1.6 (2.0 – 7.4)	4.6 ± 1.2 (2.4 – 6.3)
	% of total myelinated LTMR input onto inhibitory PV dendrites	28.5 ± 8.6 (12.5 – 41.9)	35.2 ± 3.6 (28.2 – 40.2)

Table S3 (Relates to Figure 3). Summary of Aβ- and Aδ-hair LTMR innervation of inhibitory PV cells. Aβ- and Aδ-hair afferent terminals were labelled in Split^{Cre};Ai34 and TrkB^{CreER};Ai35 mice, respectively (n = 3 mice per group), and all myelinated LTMR inputs were labelled by VGLUT1 immunoreactivity. Inhibitory PV cells were identified by co-expression of PV and Pax2 immunoreactivity. Values are given as mean ± SEM (range).

		Naïve	SNI
Somata	# of PV inhibitory synapses / PKCγ soma	4.1 ± 0.5 (3.1 – 4.7)	3.9 ± 0.5 (2.9 – 4.6)
	# of non-PV inhibitory synapses / PKCγ soma	16.1 ± 1.3 (14.0 – 18.6)	16.0 ± 1.6 (14.3 – 19.2)
	% total inhibitory input onto PKCγ somata derived from PV inhibitory synapses	19.1 ± 1.4 (17.5 – 21.9)	17.7 ± 2.3 (14.4 – 22.2)
Dendrites	# of PV inhibitory synapses / 100μm PKCγ dendrite	7.1 ± 1.1 (5.3 – 9.1)	6.0 ± 0.4 (5.3 – 6.7)
	# of non-PV inhibitory synapses / 100μm PKCγ dendrite	22.1 ± 2.5 (17.8 – 26.4)	24.3 ± 3.2 (19.7 – 30.4)
	% total inhibitory input onto PKCγ dendrites derived from PV inhibitory synapses	24.4 ± 2.4 (21.0 – 29.0)	20.2 ± 2.4 (15.5 – 23.7)
	Total dendritic length analysed / PKCγ cell (μm)	168.0 ± 21.4 (62.6 - 406.3)	171.1 ± 24.1 (51.0 – 328.5)

Table S4 (Relates to Figure 6 and Figure S3). Summary of inhibitory innervation of PKCγ cells in naive and SNI mice. n = 3 mice per group. For analysis of inputs onto PKCγ somata, 63 cells per group were analysed (21 cells per animal per group). For analysis of inputs onto PKCγ dendrites, a subset of these cells were partially reconstructed (n = 23 cells for naïve mice, range 6-10 cells per animal; 21 cells for SNI mice, 7 cells per animal). No significant differences were detected between naive and SNI mice for any of the parameters listed (see main text for details). Values are given as mean ± SEM (range).
